# Superconductivity from a melted insulator in Josephson junction arrays

**DOI:** 10.1038/s41567-023-02161-w

**Published:** 2023-08-10

**Authors:** S. Mukhopadhyay, J. Senior, J. Saez-Mollejo, D. Puglia, M. Zemlicka, J. M. Fink, A. P. Higginbotham

**Affiliations:** IST Austria, Klosterneuburg, Austria

**Keywords:** Quantum simulation, Superconducting properties and materials

## Abstract

Arrays of Josephson junctions are governed by a competition between superconductivity and repulsive Coulomb interactions, and are expected to exhibit diverging low-temperature resistance when interactions exceed a critical level. Here we report a study of the transport and microwave response of Josephson arrays with interactions exceeding this level. Contrary to expectations, we observe that the array resistance drops dramatically as the temperature is decreased—reminiscent of superconducting behaviour—and then saturates at low temperature. Applying a magnetic field, we eventually observe a transition to a highly resistive regime. These observations can be understood within a theoretical picture that accounts for the effect of thermal fluctuations on the insulating phase. On the basis of the agreement between experiment and theory, we suggest that apparent superconductivity in our Josephson arrays arises from melting the zero-temperature insulator.

## Abstract (web summary)

Predictions of a quantum superconductor–insulator transition in Josephson junction arrays are not always borne out by experiments. Unexpectedly large thermal effects may explain why.

## Main

Quantum phase transitions typically result in a broadened critical or crossover region at non-zero temperature^1^. Josephson arrays are a model of this phenomenon^2^, exhibiting a superconductor–insulator transition at a critical wave impedance^3–13^, and a well-understood insulating phase^14,15^. Yet high-impedance, one-dimensional arrays used in quantum computing^16–19^ and metrology^20^ apparently evade this transition, displaying superconducting behaviour deep into the nominally insulating regime^21^. The absence of critical behaviour in such devices is not well understood. Here we show that, unlike the typical quantum-critical broadening scenario, in one-dimensional Josephson arrays temperature dramatically shifts the critical region. This shift leads to a regime of superconductivity at high temperature, arising from the melted zero-temperature insulator. Our results quantitatively explain the low-temperature onset of superconductivity in nominally insulating regimes, and the transition to the strongly insulating phase. We further present an understanding of the onset of anomalous-metallic resistance saturation^22^. This work demonstrates a non-trivial interplay between thermal effects and quantum criticality. A practical consequence is that, counterintuitively, the coherence of high-impedance quantum circuits is expected to be stabilized by thermal fluctuations.

Josephson-array superinductors are characterized by a Josephson energy *E*_J_, junction charging energy *E*_C_ and ground charging energy *E*_g_ (ref. ^17^). These parameters must be chosen to deliver high inductance while keeping the superfluid phase stiffness large enough to resist phase slips. A common experimental strategy is to minimize the single-junction rate for quantum phase slips, $y\propto {{\mathrm{e}}}^{-4\sqrt{2{E}_{{\mathrm{J}}}/{E}_{{\mathrm{C}}}}}$ (refs. ^23–26^). However, for high-impedance arrays, the phase-slip rate is always renormalized towards infinity as temperature goes to zero^13,27^, resulting in insulating behaviour. Our key insight is that long superinductors avoid this fate by operating above the melting point of the insulating phase, where the low-temperature renormalization has yet to occur, and that this results in apparent superconducting behaviour. This effect quantitatively explains the presence of superconducting behaviour, resistance saturation and the transition to strongly insulating regimes in superinductors.

**Fig. 1:**
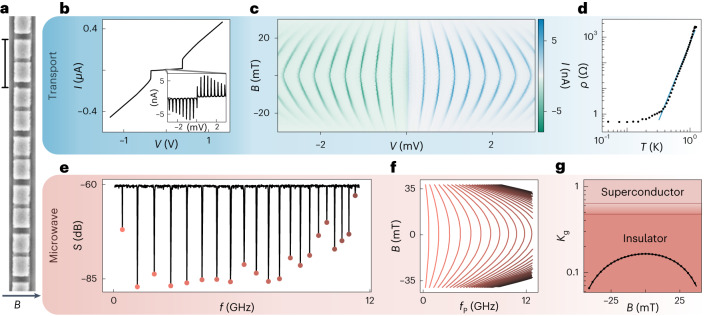
Device, transport and microwave measurement techniques. **a**, Scanning electron micrograph of a small segment of the one-dimensional Josephson array. Left scale bar, 1.5 μm. The arrow indicates the direction of the magnetic field *B*. **b**, Current *I* versus source–drain bias voltage *V*. Inset: small-scale current peaks over a narrower voltage range. **c**, Current *I* versus bias *V* and magnetic field *B* over a bias range similar to the inset in **b**. **d**, Differential resistance per junction (specific resistance) *ρ* versus cryostat temperature *T* measured at *V* = 0 and *B* = 0. The blue line shows the power-law fit. *ρ* reflects the resistance associated with the zero-bias superconducting branch, found by measuring the two-probe resistance, subtracting off four-probe-measured line resistance and then dividing by number of junctions. **e**, Two-tone microwave spectroscopy. Probe-tone transmission *S* versus pump-tone frequency *f*, with probe-tone frequency fixed to resonance at approximately 6.11 GHz. Extracted plasma-mode resonant frequencies *f*_P_ indicated by coloured markers. **f**, Evolution of measured plasma-mode frequencies *f*_P_ with applied magnetic field *B*. **g**, Superfluid phase stiffness ${K}_{{\mathrm{g}}}=\sqrt{{E}_{{\mathrm{J}}}/(2{E}_{{\mathrm{g}}})}$, experimentally inferred from plasma modes in **f**, versus *B* (black line; black markers show every fifth data point). Theoretically expected superconducting and insulating regimes are labelled, and demarcated by a band covering the clean^2^ and dirty^27^ limits. Source data

Two nearly identical devices are studied: one galvanically coupled to electrical leads permitting the measurement of resistance, and one capacitively coupled to microwave transmission lines permitting the measurement of plasma modes^17,21^. Both devices consist of an array of approximately 1,220 Josephson junctions fabricated using electron-beam lithography and a standard shadow evaporation process on high-resistivity silicon substrates (Fig. 1a). For nanofabrication reasons, the array islands have alternating thickness, which, in the presence of magnetic field, ideally gives rise to an alternating gap structure while maintaining a uniform Josephson energy throughout the chain. At zero magnetic field, each junction has nominally identical *E*_J_/*h* ≈ 76 GHz, *E*_g_/*h* ≈ 1,400 GHz and *E*_C_/*h* ≈ 5 GHz, where *h* is Planck’s constant. These parameters are determined from analysing microwave (*E*_J_ and *E*_g_) and transport (*E*_C_) measurements with several consistency checks, as described below and in Supplementary Section I.

The working principle of the experiment is to leverage the complementary strengths of low-frequency electrical transport and microwave-domain circuit quantum electrodynamics. These techniques differ by nine orders of magnitude in characteristic frequency, and combine to give access to both the scaling behaviour, associated with low energies (transport), and the microscopic system parameters, associated with high energies (microwave).

In the transport device, a linear current (*I*)–voltage (*V*) characteristic at large applied voltage bias gives way to a high-resistance region below a critical voltage, whose value is approximately given by the number of junctions *N* times twice the superconducting gap *Δ* (Fig. 1b). Over a smaller range of applied voltage a series of evenly spaced current peaks are observed with an apparent supercurrent at zero bias (Fig. 1b inset). The transport mechanisms associated with the high-bias current peaks are not clearly understood, although their locations are suggestive of a picture of successive voltage drops across *N* voltage-biased Josephson junctions, with low current on the quasiparticle branches and high current when bias is a multiple of 2*Δ*/*e*, where *e* is the electron charge (see Supplementary Section VI and refs. ^11,20^).

Increasing magnetic field *B* parallel to the chip plane suppresses supercurrent, suggesting a field-driven transition from a superconducting to an insulating state (Fig. 1c). The spacing between current peaks also decreases with *B*, indicating a reduction in the superconducting gap with magnetic field. In the strongly superconducting regime (*B* = 0), zero-bias differential resistance per junction (specific resistance) associated with the superconducting branch decreases dramatically with cryostat temperature (Fig. 1d), dropping over more than three decades before saturating to a low value of <1 Ω per junction. The observed resistance saturation is not compatible with finite-size effects; we estimate that the device length exceeds the thermal length by an order of magnitude at 150 mK. The precipitous drop in specific resistance at low temperature and supercurrent features in nonlinear transport give a preliminary indication of the dominance of superconducting behaviour. We will develop a framework for understanding the behaviour of specific resistance in detail, but first turn to the complementary use of microwave techniques to independently determine the system parameters.

Microwave spectroscopy is performed by monitoring the transmission of a weak probe signal while the frequency of a strong pump tone is varied^17^ (see Supplementary Section I.B for details). A series of sharp dips are observed in probe-tone transmission *S* (Fig. 1e), corresponding to plasma modes of the array. The plasma modes are evenly spaced at low frequency, reflecting the speed of light and length of the array, and are clustered at high frequency due to proximity with the single-junction plasma frequency. A simple fitting procedure allows extraction of the array parameters from the microwave data. Performing two-tone spectroscopy as a function of field (Fig. 1f), the array parameters *E*_g_, *E*_C_ and *E*_J_(*B*) are fully characterized as a function of magnetic field. With these values fixed experimentally, it is straightforward to perform parameter-free comparisons with the theory of the superconductor–insulator transition in one dimension. Of particular importance is the superfluid phase stiffness, ${K}_{{\mathrm{g}}}=\sqrt{{E}_{{\mathrm{J}}}/(2{E}_{{\mathrm{g}}})}$, which quantifies the ability of the array to resist phase slips. Below a critical value of *K*_g_, theory predicts that phase slips dominate and insulating behaviour emerges^2,27^.

Performing this comparison (Fig. 1g) reveals that the array’s phase stiffness is as much as an order of magnitude below the critical value for insulating behaviour^2,27^, in contrast to the observed superconducting behaviour in transport. Thus, combining the transport and microwave measurements reveals an apparent conflict with basic expectations for the superconductor–insulator phase transition. Resolving this conflict is the central subject of this work.

**Fig. 2:**
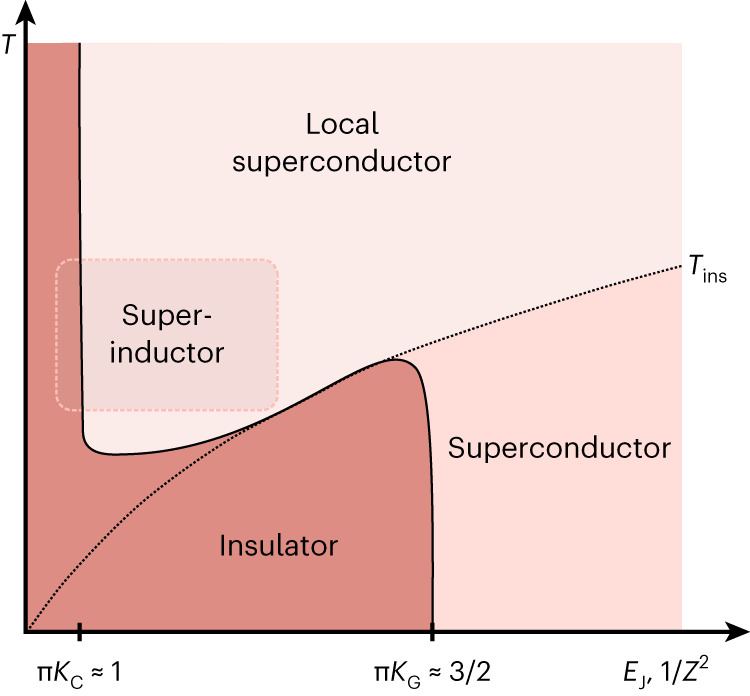
Proposed phase diagram. Map of superconducting and insulating states as a function of Josephson energy *E*_J_ and temperature *T*. The wave impedance $Z=\hslash /(4{e}^{2}){K}_{{\mathrm{g}}}^{-1}$ satisfies 1/*Z*^2^ ∝ *E*_J_ for constant *E*_g_. The dashed line marks the boundary between long-range and short-range behaviour, *T*_ins_, given by equation (1). Below *T*_ins_, the physics is governed by the long-range phase stiffness *K*_g_ with a superconductor–insulator transition at π*K*_g_ ≈ 3/2. Above *T*_ins_, physics is governed by the short-range phase stiffness *K*_C_ with a superconductor–insulator transition as π*K*_C_ ≈ 1. The solid black curve traces the crossover from local to global superconductor–insulator transition. The outlined box indicates superinductance region probed in this experiment.

The theoretical picture for understanding our observations was developed in ref. ^13^. Near the superconductor–insulator transition, thermal fluctuations are controlled by the timescale *τ* = *h*/*k*_B_*T* and the associated thermal length *l*_th_ = *vτ*, where *v* is a characteristic velocity with dimensions of unit cells per time, *k*_B_ is the Boltzmann constant and *T* is temperature. *l*_th_ must be compared with the electrostatic screening length in units of unit cells, ${{\varLambda }}=\sqrt{{E}_{{\mathrm{g}}}/{E}_{{\mathrm{C}}}}$. At high temperature (*l*_th_ < *Λ*), the system is governed by the local superfluid phase stiffness, ${K}_{{\mathrm{C}}}=\sqrt{{E}_{{\mathrm{J}}}/(2{E}_{{\mathrm{C}}})}$. In contrast, at low temperature (*l*_th_ > *Λ*), the system is governed by the long-range superfluid phase stiffness *K*_g_, as assumed by standard theories of the superconductor–insulator transition. In the superinductor limit superconductivity is locally stiff, *K*_C_ ≫ *K*_g_, which results in a curious regime of local superconductivity that arises from a melted *T* = 0 insulator (Fig. 2). The ‘melting point’ of the insulator, above which local superconductivity dominates, is

1 $${T}_{{{{\rm{ins}}}}} \approx \sqrt{2{E}_{{\mathrm{J}}}{E}_{{\mathrm{C}}}}/{{\varLambda }}.$$

In the locally superconducting regime, we find that the high-temperature behaviour of the specific resistance follows a power law

2 $$\rho ={\rho }_{0}{\big(T/{T}_{{\mathrm{p}}}\big)}^{\uppi {K}_{{\mathrm{C}}}-1},$$

where${T}_{{\mathrm{p}}}=\sqrt{2{E}_{{\mathrm{J}}}{E}_{{\mathrm{C}}}}/{k}_{{\mathrm{B}}}$ is the plasma temperature and *ρ*_0_ is the specific resistance at *T* = *T*_p_ (see Supplementary Section XV for further discussion). Local superconductivity gives way to insulating behaviour when π*K*_C_ ≈ 1. In contrast, in the low-temperature limit the power-law exponent is 2π*K*_g_ − 3, which yields the typical superconductor–insulator prediction π*K*_g_ ≈ 3/2.

**Fig. 3:**
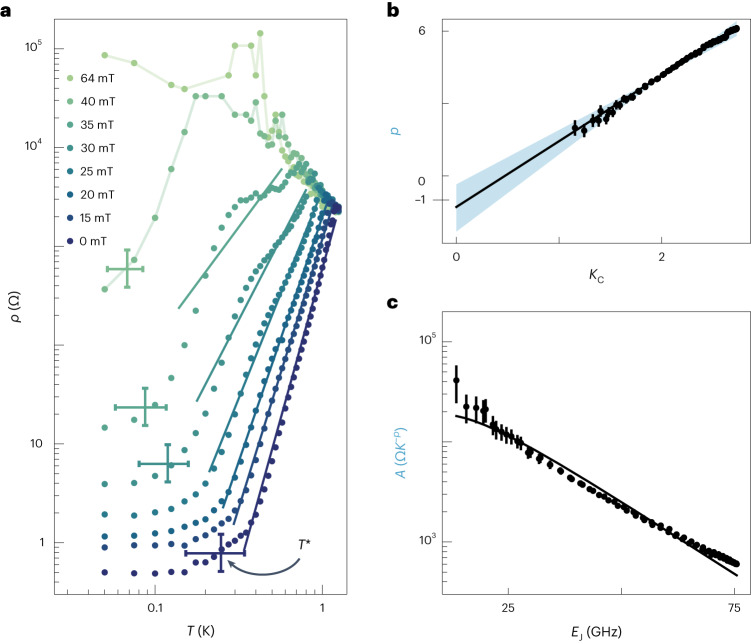
Power-law nature of local superconductivity. **a**, Zero-bias differential specific resistance *ρ* as a function of temperature *T*, at various magnetic fields. Solid lines are fits to power-law expression *ρ* = *AT*^*p*^. T* is the crossover temperature from power law to saturation behaviour, extracted from when the specific resistance goes a fixed percentage above its minimum value. This percentage range was chosen to reflect the width of the crossover region, and to pass a self-consistency check with extrapolated power-law fits (Supplementary Section XI.G). Vertical (lower, upper) error limits are (5%, 150%) above minimum resistance, the centre value is 77.5% above minimum and the horizontal error limits are the temperatures corresponding to the (lower, upper) resistance values. **b**, Exponent *p* from power-law fits to transport data in **a** versus the local phase stiffness *K*_C_ from microwave measurements. The solid line is a linear fit. The shaded blue region depicts range of linear fits obtained by repeating the entire analysis with different low-temperature cut-offs for the power-law fits (Supplementary Section XI.E). The centre of the shaded region is a low-temperature cut-off 0.215*T*_P_, which was used throughout the analysis. **c**, Amplitude *A* from power-law fits to transport data versus Josephson energy *E*_J_ from microwave measurements. The error bars in **b** and **c** are standard errors from the power-law fits shown in **a**. Source data

The experimentally studied devices have, at *B* = 0, π*K*_g_ < 1 < π*K*_C_ and *T*_ins_ ≈ 70 mK, giving an initial suggestion that they are governed by local superconductivity even at low temperatures. This hypothesis can be tested by comparing experimental measurements of temperature-dependent specific resistance, *ρ*(*T*), with the predicted power law in equation (2). As shown in Fig. 3a, increasing magnetic field weakens the temperature dependence of the specific resistance, eventually giving way to a superconductor–insulator transition at high magnetic field (*B* ≈ 44 mT). Fitting each specific resistance curve to a power law *ρ* = *AT*^*p*^ indicates that, on the superconducting side, the exponent *p* steadily decreases with field (*A* is the power-law amplitude). Comparing *p* from the transport measurements with the local phase stiffness *K*_C_ inferred from microwave measurements reveals a linear behaviour (Fig. 3b) with a slope of 2.7 ± 0.5 and an intercept of −1.3 ± 1.0, in agreement with the predicted slope π and intercept −1 for local superconductivity from equation (2), *p* = π*K*_C_ − 1. The parameter uncertainties are propagated from systematic bands in Fig. 3b (for details, see Supplementary Section XI.E). The amplitude dependence on *E*_J_ (Fig. 3c) is also in reasonable agreement with the prediction of equation (2), $A={\rho }_{0}/{T}_{{\mathrm{p}}}^{\,\uppi {K}_{{\mathrm{C}}}-1}$, with a single free parameter, *ρ*_0_ = 3.98 ± 0.02 kΩ, which is slightly larger than the single-junction tunnel resistance 2.35 kΩ, reflecting the fact that, at the plasma temperature, the observed chain resistance is higher than its normal-state value.

Figure 3b,c shows the remarkable predictive power of equation (2) in the low-field, superinductor regime. At higher magnetic fields, as the superconductor–insulator transition is approached, power-law behaviour is interrupted by a shoulder-like feature, violating equation (2). The shoulder could reflect the relevance of disorder near the superconductor–insulator transition, which is expected to result in complex structure in *ρ*(*T*) as phase slips become progressively more important^13^. A second possible origin is suppression of the superconducting gap due to magnetic field which, particularly at elevated temperature, could cause violations of the simple rotor approximation on which equation (2) is based (see Supplementary Section X for discussion of magnetic-field scales).

**Fig. 4:**
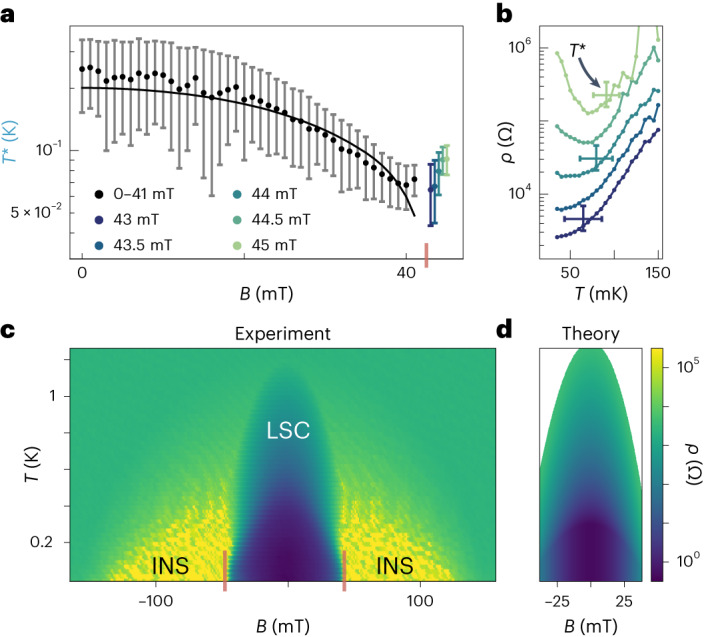
Crossover physics and phase diagram. **a**, Crossover temperature T* versus magnetic field *B*. The black line is T* ∝ *T*_ins_ with a proportionality constant of 2.5. Coloured markers indicate crossover temperature at higher fields from dataset in **b**. The red vertical line indicates π*K*_C_ = 1, where local superconductor–insulator transition is expected. Vertical error bars derived from the percentage above minimum resistance (5%, 150%) for the (lower, upper) ranges, as shown in **b** and Fig. 3a. **b**, Zero-bias differential specific resistance *ρ* versus temperature *T* at higher magnetic fields, measured with higher lock-in excitation voltage and more averaging than in Fig. 3a to resolve low-temperature behaviour (Supplementary Section X.B). T* and its error bars are extracted using the same method as in Fig. 3a. Vertical (lower, upper) error limits are (5%, 150%) above minimum resistance, the centre value is 77.5% above minimum and the horizontal error limits are the temperatures corresponding to the (lower, upper) resistance values. **c**, *ρ* versus temperature *T* and magnetic field *B*. The dome of local superconductivity (LSC) and wings of insulating behaviour (INS) are labelled. The red vertical lines indicate π*K*_C_ = 1, where local superconductor–insulator transition is expected. **d**, Calculated specific resistance *ρ* as a function of temperature *T* and magnetic field *B*. Source data

The boundaries of local superconductivity can also be understood within the picture of Fig. 2. At low temperatures, the experimentally observed power-law behaviour in specific resistance saturates at a crossover temperature T* (indicated in Fig. 3a). The crossover temperature decreases with magnetic field, as shown in Fig. 4a, qualitatively agreeing with the expected square-root dependence for T* ∝ *T*_ins_, albeit within large error bars due to uncertainty in the extraction of T*. This agreement supports the view that the low-temperature saturation is in fact a crossover into the insulating state. At high magnetic fields corresponding to π*K*_C_ > 1, T* instead increases with magnetic field (Fig. 4b), consistent with a superconductor–insulator transition entering into the non-perturbative insulating regime of ref. ^13^, where the phase-slip rate, $\propto {e}^{-4\sqrt{2{E}_{{\mathrm{J}}}/{E}_{{\mathrm{C}}}}}$, is no longer small. We caution that the experimental interpretation of T* is complicated for two reasons. First, although we have performed normal-state electron thermometry and radiation thermometry and found that all characteristic temperatures are below T*, thermalization at the actual superconductor–insulator transition is difficult to verify directly. Second, different metrics for T* can give quantitatively different scaling with *B*, although the decreasing trend predicted by equation (1) and upturn at high field are robust features visible even in the raw data.

The complete behaviour of the Josephson array can be summarized by measuring a specific resistance ‘phase diagram’. Mapping zero-bias differential specific resistance as a function of magnetic field and temperature reveals a characteristic dome at low field, already identified from the power-law analysis as a local superconductor, giving way to a high-specific-resistance insulating phase as the magnetic field is increased (Fig. 4c). The low-temperature boundary between superconducting and insulating states occurs at π*K*_C_ ≈ 1, as expected. After the high-field boundary of the strongly insulating regime (~145 mT), temperature-dependence and current–voltage characteristics resemble a normal array of metallic islands^28^.

The local superconducting dome and its boundaries can be quantitatively modelled as follows. The thermal boundary of the dome is *T* = *T*_p_, the upper cut-off scale of our renormalization-group approach^13^. For *αT*_ins_ < *T* < *T*_p_, equation (2) applies, with *ρ*_0_ from Fig. 3c. For *T* < *αT*_ins,_ specific resistance saturates due to a crossover into the insulating regime, and would presumably increase at lower, experimentally inaccessible, temperatures. The constant *α* = 5, which tunes the crossover to insulating behaviour in the model, is fixed from the experimentally observed saturation of specific resistance at *B* = 0 and is in reasonable agreement with the constant found in Fig. 4a. For sufficiently large *B* one approaches π*K*_C_ = 1, which sets the magnetic-field boundaries of the dome. Calculating *ρ* according to this procedure results in a local superconducting dome in satisfactory agreement to the experiment (Fig. 4d). This gives evidence that the presence of local superconductivity, and its proximity to insulating phases, is well understood.

Summarizing, by combining transport and microwave measurements, we have uncovered strong evidence for a locally superconducting state in Josephson arrays arising from a *T* = 0 insulator. This resolves the problem of apparent superconductivity in nominally insulating regimes, and clarifies where superconductor–insulator transitions are actually observed in experiment. Our work sheds light on the observation of the high-quality microwave response in the nominally insulating regime of superinductors^21^, suggesting effects in addition to high-frequency mechanisms that have been previously discussed^26,29,30^. Such devices operate near the ‘sweet spot’ *T* ≈ *T*_ins_ where temperature is low enough for well-developed local superconductivity, yet high enough to melt insulating behaviour. As a consequence, we suggest that the performance of some high-impedance quantum devices^18,19,31^ is actually improved by thermal fluctuations. It is also interesting to consider whether experimental studies of insulating behaviour in resistively shunted Josephson junctions^32–35^ could be understood by carefully considering the role of non-zero temperature, finite-size or non-perturbative effects^36^.

Viewed from the broader perspective of response functions near quantum criticality, we have demonstrated a rare example where the thermal fluctuations with timescale *τ* = *h*/*k*_B_*T* can be quantitatively traced through to experimentally measured specific resistance^13^. This does not result in an effectively Planckian scattering (Supplementary Section XII.B), as was recently observed in a different superconductor–insulator system^37^. It is also interesting to note that our saturating specific resistance curves empirically bear a strong resemblance to the anomalous-metallic phase in two-dimensional systems^22^. In our case, saturation is understood as a crossover effect towards insulating behaviour. It would be interesting to perform a similar experimental programme on a known anomalous-metallic system to test whether saturation can be understood as a similar crossover effect.

## Online content

Any methods, additional references, Nature Portfolio reporting summaries, source data, extended data, supplementary information, acknowledgements, peer review information; details of author contributions and competing interests; and statements of data and code availability are available at 10.1038/s41567-023-02161-w.

### Supplementary information

Supplementary Information Supplementary Sections I–XV, Figs. 1–20 and Tables I–V.

Supplementary Data and Code 1 Contains all data in the paper, including supplement, serialized into text files, and Python scripts that recreate all figures from the text files.

### Source data

Source Data Fig. 1 All data used in Fig. 1 in text files. See Supplementary Data and Code 1 for plotting code.

Source Data Fig. 3 All data used in Fig. 3 in text files. See Supplementary Data and Code 1 for plotting code.

Source Data Fig. 4 All data used in Fig. 4 in text files. See Supplementary Data and Code 1 for plotting code.

## Data Availability

Source data are provided with this paper. Source data for Supplementary Figs. 1–20 are available in Supplementary Data and Code 1. Additional data are available from the corresponding author upon request (refs. ^38–51^). Fabrication, measurement and analysis methods are described in Supplementary Information, which contains refs. ^38–51^.
