# Supplementary material for: Superconductivity from a melted insulator in Josephson junction arrays
Source: Nat Phys. 2023 Aug 10;19(11):1630–5. doi: 10.1038/s41567-023-02161-w (PMC10635826; doi:10.1038/s41567-023-02161-w)
Supplement: Supplementary file 1 — Supplementary Sections I–XV, Figs. 1–20 and Tables I–V. [file 41567_2023_2161_MOESM1_ESM.pdf]

nature physics

Article https://doi.org/10.1038/s41567-023-02161-w

Superconductivity from a melted insulator in Josephson junction arrays

In the format provided by the authors and unedited

Supplementary information for "Superconductivity from a melted insulator in Josephson junction arrays"

S. Mukhopadhyay,[1,*] J. Senior,[1,*] J. Saez-Mollejo,[1] D. Puglia,[1] M. Zemlicka,[1] J.M. Fink,[1] and A.P. Higginbotham[1,†]
[1] *IST Austria, Am Campus 1, 3400 Klosterneuburg, Austria*

CONTENTS

I. Extraction of chain parameters 2
A. Josephson energy interpolation 3
B. Two-tone spectroscopy 3
C. Chain and junction parameters 4

II. Schematic of the chip 5

III. Device setup and connections 6

IV. Nanofabrication 7

V. Current-peak spacings 8

VI. Qualitative picture of finite-bias transport 9

VII. Finite-bias differential conductance 10

VIII. Base electron temperature 11

IX. Comparing high-bias transport and zero-bias phase diagram 12

X. Magnetic-field tuning 12

XI. Measurement and analysis checks 13
A. Lock-in excitation 14
B. Higher lock-in excitation for Fig. 4b 14
C. Lower-field resistance upturn 15
D. Voltage Offsets 16
E. Power law fits 17
F. Comparison of experiment with infrared theory 18
G. Power-law extraction of crossover temperature 19
H. Low wave-number resonant frequencies 20

XII. Additional analysis 21
A. Scaling 21
B. Planckian limit 22

XIII. Comparison with previous work 22

XIV. Outlook for accessing infrared physics 23

XV. Theory 23
A. Boundaries in theoretical phase diagram 24

---

* Equal contribution
† andrew.higginbotham@ist.ac.at

I. EXTRACTION OF CHAIN PARAMETERS

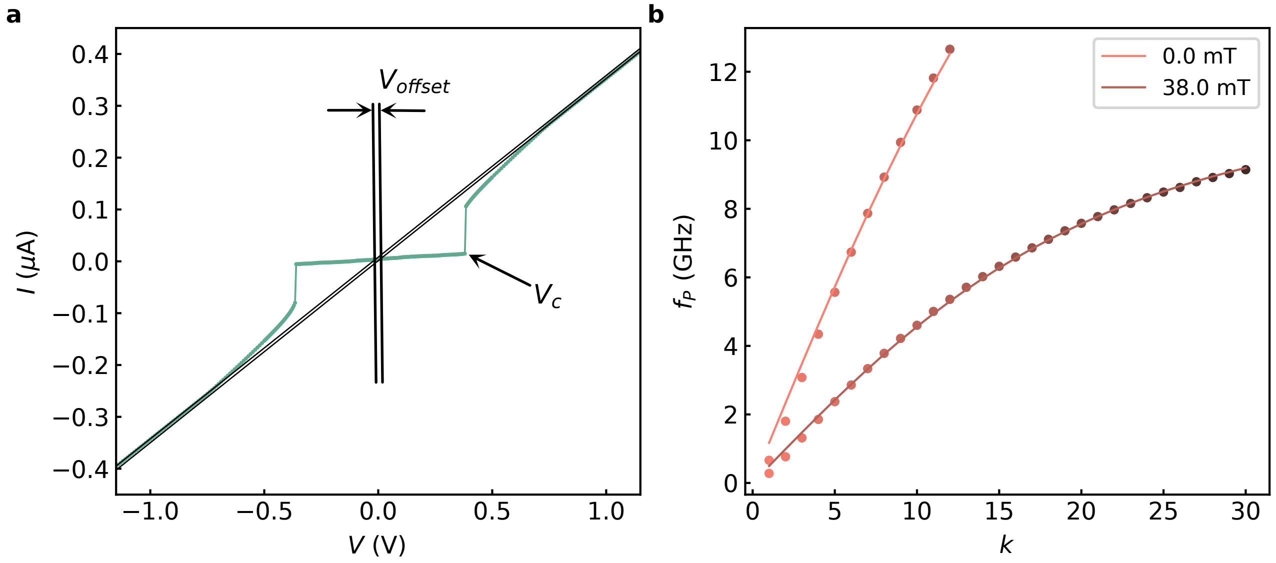

Figure S1. **Extraction of chain parameters $\mathbf{E_C, E_J, E_g}$. a,** Measured current I versus applied voltage V. Linear fits to the high bias features extract E_C, E_J. V_c is the critical voltage. **b,** Extracted resonant peak frequency f_P versus mode number k. The curves are fit from the dispersion relation, which yields E_g, and E_J as a function of magnetic field.

In the transport device, a voltage offset V_{offset} is extracted by extrapolating the linear parts of the current-voltage characteristic down to zero bias (Fig. S1a). E_C is then inferred from V_{offset} using [15]

$$E_C = 4eV_{\text{offset}}/N, \tag{S1}$$

which gives the value quoted in the main text.

Once E_C is fixed from transport, microwave measurements are used to determine E_g and $E_J(B)$. In the limit of large number of junctions N, the dispersion relation [13, 17] for plasma-mode resonant frequency $\omega_{P,k}$ is

$$\omega_{P,k} = \omega(k)/\sqrt{1 + (\omega(k)/\omega_p)^2}, \tag{S2}$$

where $\hbar\omega_p = \sqrt{2E_J E_C}$ and $\hbar\omega(k) = \pi k\sqrt{2E_J E_g}/N$, with E_J, E_C and E_g defined as in Table SIV. Fitting Eq. S2 to the experimental data, as in Fig. S1b, yields E_g and $E_J(B)$. Sample values determined with this method are presented in Fig. S2, Table SII.

Two independent checks are available on the extracted system parameters. The charging energy E_C can be estimated from geometry and the nominal specific capacitance of our Josephson junctions. Josephson energy can be estimated with the Ambegaokar-Baratoff relation [38]

$$E_J = N\Delta h/(8e^2 R_N), \tag{S3}$$

where N is the number of junctions and Δ is the superconducting gap of Aluminum. These independent checks on E_C and E_J are shown in Table SIII.

A. Josephson energy interpolation

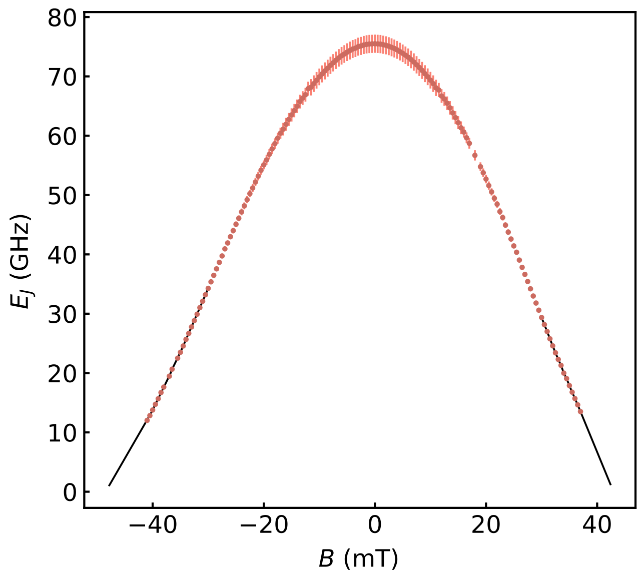

Figure S2. $\mathbf{E_J(B)}$ **interpolation.** Josephson energy E_J, determined from fitting dispersion curves, as a function of the magnetic field B (points, orange error bars). Error bars are the standard error from fits of Eq. S2 (see Fig. S1 and associated discussion). Black lines represent a linear interpolating function used at higher magnetic fields used to estimate B^{ins}, see Sec. IX for further details.

The field-driven superconductor-insulator transition in our system is expected to occur at a magnetic field B^{ins}. As discussed in Sec. IX, B^{ins} is determined by numerically solving $\pi K_c(B^{\text{ins}}) = 1$. To accomplish this we interpolate $E_J(B)$ linearly out to B^{ins}, which is justified by the smooth, linear behavior observed in E_J over similar field ranges, as shown in Fig. S2.

B. Two-tone spectroscopy

Fig. 1e is two-tone spectroscopic data at $B = 30$ mT. For Fig. 1f, similar data were acquired at each magnetic field, automatically adjusting the probe-tone frequency at each magnetic field to be at maximum transmission within the measurement band (*e.g.* 6.11 GHz at 30 mT).

Two-tone spectroscopy utilizes the slight non-linearity of a Josephson junction array, arising from a quartic expansion of the Josephson potential. This results in interaction between different plasma modes of the array, known as the cross-Kerr effect. This interaction causes dispersive shift δ_{ij} of a mode ω_i, linearly dependent on the photon number occupation n_j of a mode ω_j [21, 39, 40]. Utilizing this property, the entire spectrum of the JJ array can be mapped out at given magnetic field.

The following experimental procedure was adopted. Using a Vector Network Analyzer (VNA), the resonant mode frequency with maximum transmittance was first mapped out as a function of applied magnetic field. Then, at a fixed field, the VNA was used to generate a weak probe signal at the mode frequency ω_i of maximum transmittance. The transmission of the probe tone was monitored while sweeping the frequency of a second, strong pump tone. When the pump frequency matched with a plasma mode resonance ω_j, this changed the measured transmission at the probe frequency due to the dispersive shift δ_{ij} from the cross-Kerr effect, causing the dips in Fig. 1e. Repeating the procedure at different applied magnetic fields and extracting the mode frequencies resulted in the data in Fig. 1f.

C. Chain and junction parameters

Parameter	Notation	Microwave	Transport
Number of junctions	N	1227	1217
Chain length	L	912 μm	905 μm
Junction Area	A	$0.28 \pm 0.01\ \mu\text{m}^2$	

Table SI. **Chain and junction geometry.** Chain length and number of junctions refer to the designed values. Geometry of a junction (or the chain) is estimated from SEM imaging. For details refer to section *Nanofabrication.*

The error in A is the propagated error from estimating length and breadth of a single junction from a SEM image.

Parameter (@ $B = 0$ T)	Notation	Value	Method
Charging energy	E_C/h	5.11 ± 0.04 GHz	Eq. S1
Charging energy to ground	E_g/h	1390 ± 40 GHz	Fig. S1b
Josephson energy	E_J/h	75.5 ± 1.5 GHz	Fig. S1b
Normal state resistance of chain	R_N	2857.11 ± 0.08 kΩ	Fig. S1a
Critical voltage of chain	V_c	0.374 ± 0.001 V	Fig. S1a

Table SII. **Extracted parameters.** For calculations discussed in text, E_J and E_g inferred from microwave measurements are used, whereas E_C inferred from transport measurements are used.

The error in E_g is the standard error in the values of E_g inferred from fits to the dispersion curves at high magnetic fields. The error in E_J is the standard error from fit to the dispersion curve at $B = 0$ T. The error in E_C, R_N and V_c is the difference between charging energies, high-bias resistances and critical voltages inferred from up and down I-V sweeps.

The following table serves as a cross-check of the junction parameters mentioned in Table SII above:

Parameter	Value	Method
Charging energy	6.1 ± 0.2 GHz	$(2e)^2/(2C_s \times A)/h$
Josephson energy	54.73 ± 0.06 GHz	Eq. S3

Table SIII. **Independent checks on extracted parameters.** Empirical specific capacitance of a junction $C_s = 45$ fF/μm^2 [41]. Superconducting gap of bulk Aluminum, $\Delta = 180\ \mu$eV.

The errors in the parameters of Table SIII are propagated from the errors in A (Table SI) and R_N (Table SII). Table SIV contains a list of chain/junction parameters that have been derived from the measured values presented in Table SII:

Parameter (@ $B = 0$ T)	Notation	Value	Formula
Junction capacitance	C_c	15.1 ± 0.1 fF	$(2e)^2/(2E_C)$
Junction capacitance to ground	C_g	55.9 ± 1.5 aF	$(2e)^2/(2E_g)$
Junction inductance	L_{J0}	2.16 ± 0.04 nH	$(\hbar/2e)^2/E_J$
Impedance	Z	6.2 ± 0.2 kΩ	$\sqrt{L_{J0}/C_g}$
Local superfluid phase stiffness	K_C	2.72 ± 0.04	$\sqrt{E_J/2E_C}$
Global superfluid phase stiffness	K_g	0.165 ± 0.003	$\sqrt{E_J/2E_g}$
Plasma frequency	f_P	27.7 ± 0.3 GHz	$\sqrt{2E_JE_C}/h$
Charge screening length	Λ	16.4 ± 0.2	$\sqrt{E_g/E_C}$
Bloch bandwidth	W	149 ± 3 Hz	Eq. S4

Table SIV. Chain/junction parameters inferred from Table SII.

$$W = 16(E_JE_C/\pi)^{1/2}(2E_J/E_C)^{1/4}e^{-\sqrt{32E_J/E_C}}. \tag{S4}$$

The errors for each parameter in Table SIV are propagated from corresponding errors in the parameters mentioned in Table SII. Capacitances (C_c, C_g) are calculated with charging energy being defined as the cost of accommodating

an extra Cooper pair, following the convention used in Ref. [26]. Following Ref. [42], Josephson energy E_J and the Josephson supercurrent I_s is defined as

$$E_J = \hbar I_c/2e, \tag{S5}$$
$$I_s = I_c sin(\phi), \tag{S6}$$

where ϕ is the difference in phase of the Ginzburg-Landau wavefunction in the two superconducting islands. The voltage V maintained across the junction is related to ϕ by the following relation [42]

$$\frac{d\phi}{dt} = (2eV)/\hbar. \tag{S7}$$

Differentiating equation S6 with respect to t and utilizing S7, the voltage V can be expressed as

$$V = \frac{\hbar}{2eI_c cos(\phi)} \frac{dI_s}{dt}. \tag{S8}$$

Since $V = LdI/dt$, the phase dependent Josephson inductance $L_J(\phi) = L_{J0}/cos(\phi)$, where [43]

$$L_{J0} = \hbar/(2eI_c). \tag{S9}$$

II. SCHEMATIC OF THE CHIP

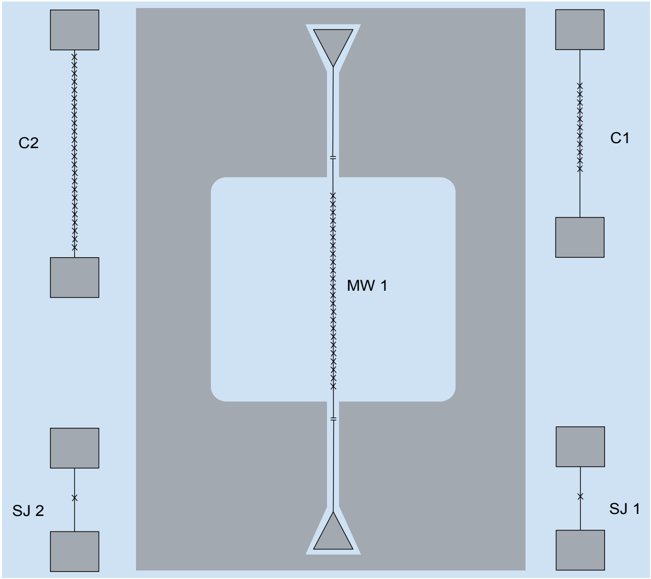

Figure S3. **Schematic of the nanofabricated chip.** Blue background represents Silicon. Grey represents Aluminum. Each cross represents a single Josephson junction.

MW1 refers to the chain of Josephson junctions capacitively coupled on either side to 50 Ω microwave launchers. C1 and C2 refer to transport chains. C2 contains nominally the same number of junctions as on the microwave chain, MW1. C1 has half as many junctions as C2. SJ1 and SJ2 are identical single junction transport devices, with the same junction geometry as on the microwave and transport chain devices. Ground plane around the array of junctions of the microwave device (MW1) has been removed with the intention of decreasing capacitance to ground.

III. DEVICE SETUP AND CONNECTIONS

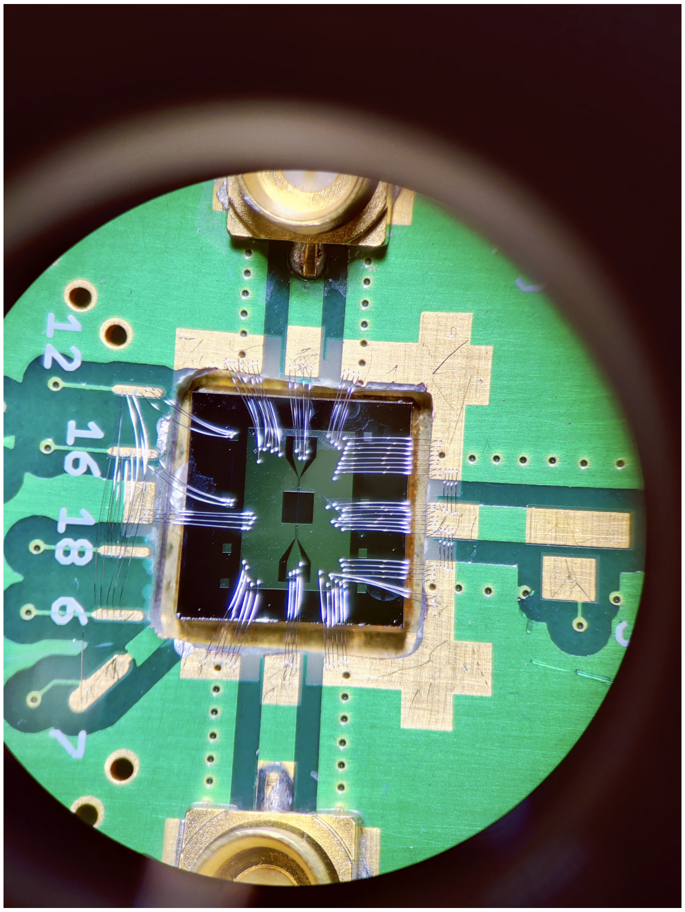

Figure S4. **Chip bonded onto PCB.** Numbers on the PCB indicate connections to DC lines for four/two probe transport measurements. SMP launchers for microwave connections can be seen partially at the top and bottom of the picture.

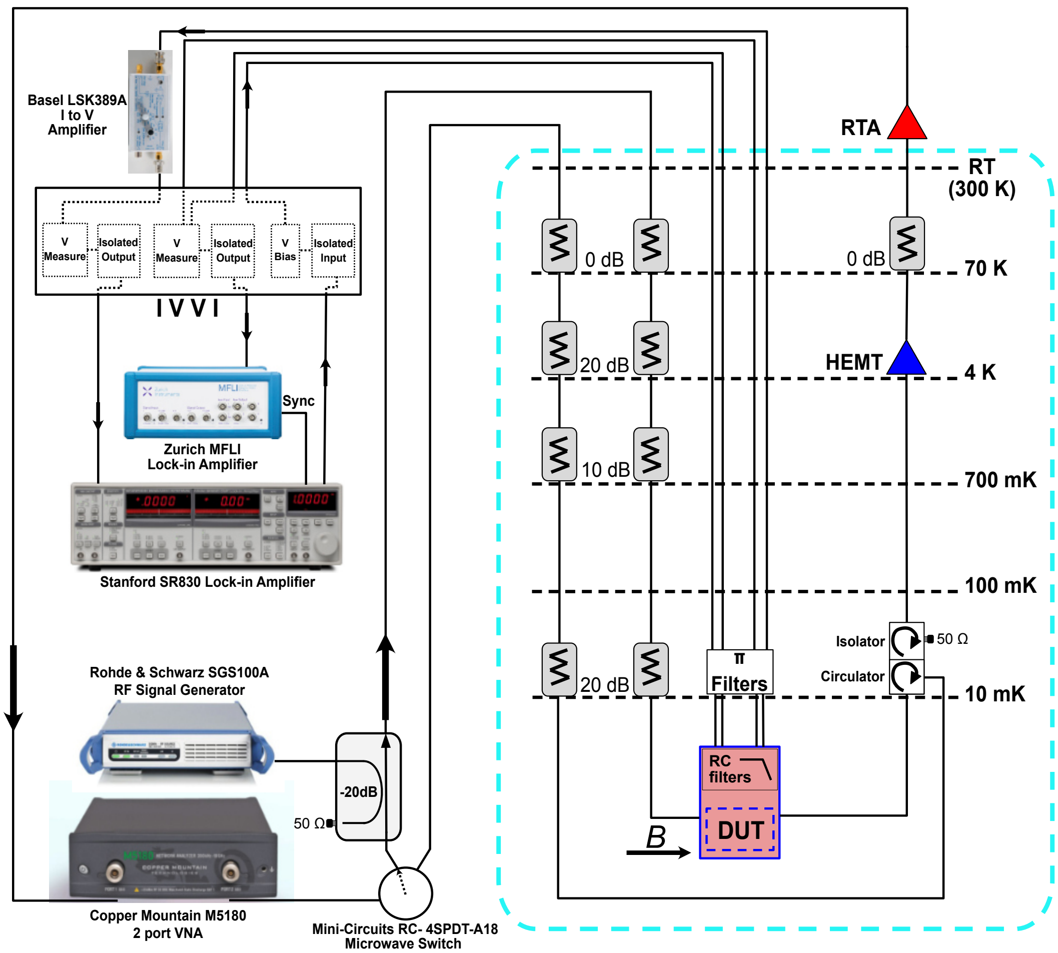

Figure S5. **The experimental setup.** The Dilution Fridge represented as cyan colored dashed boundary. The signal generator is coupled to the output port of the VNA through a RF coupler which has a 20 dB insertion loss at the coupled port. The 'resistor' symbol at each stage of the fridge represent the installed microwave cryogenic attenuators. 'RTA' refers to Room Temperature Amplifier with a gain of around 35 dB. 'HEMT' refers to High-Electron-Mobility-Transistor, which is the Low Noise Amplifier (noise ~ 2 K) with a gain of around 36 dB at 4 K. 'DUT' refers to 'Device Under Test'. 'B' indicates the direction of applied external magnetic field.

Microwave measurements are done in transmission, where the output (amplifier) line has double junction isolator attached to the mixing chamber stage of the dilution fridge. In addition, the output line has LNF HEMT attached to the 4 K stage and and another LNF amplifier attached to the room temperature stage of the cryostat. The input line has 50 dB of net attenuation with 0 dB at 70 K stage, 20 dB at 4 K stage, 10 dB at 700 mK stage and 20 dB at mixing chamber stage of the cryostat.

The DC lines are equipped with three cascaded LFCN filter boards attached to the mixing chamber stage of the dilution fridge; each filter board provides cut-off at different frequencies. The three cut-off frequency ranges are (DC – 5000 MHz), (DC – 1450 MHz) and (DC – 80 MHz). Each board has filters soldered onto them in six stages for all the DC lines. The PCB onto which the chip is bonded, has single stage low pass filtering (2 kΩ ; 47 nF) for each DC line. So, in all each DC line has nineteen stage low-pass filtering at the mixing chamber stage of the cryostat.

IV. NANOFABRICATION

- The chains were fabricated on a high resistivity Silicon substrate ($> 10^4$ Ωcm), which was diced into 7×7 mm chips, ultrasonicated in Acetone (2 min / power 4) and then in IPA (2 min / power 4).

- The chips were baked at 170 C for 3 mins before spin coating with MMA (EL-13), followed by PMMA (950k 4%). Spin coating recipe was developed such that thickness of MMA is 670 nm and of PMMA is 290 nm. The

chips were again baked at 170 C for 3 mins after each spin coating step.

- Standard e-beam lithography was done in Raith EBPG5150, after which the pattern was developed in IPA : water (3 : 1) solution for 90 secs.

- The developed chip was then subjected to electron beam evaporation with Aluminum, in a double angle shadow evaporation process in Plassys UHV MEB550S2, with an intermediate in-situ static oxidation step (5 mbar/5 mins). The evaporation was terminated with another in-situ static oxidation step (10 mbar/2 mins). Before evaporating Aluminum, the evaporation chamber was gettered with Titanium for 3 mins at 0.2 nm/sec to further bring down the pressure of the chamber. In the first evaporation step, 55 nm of Aluminum was deposited, whereas on the second step 110 nm of Aluminum was deposited, with an evaporation rate of 1 nm/sec in both steps.

- Lift-off was done using hot NMP (80 C) for 45 mins, after which the chip was successively cleaned in cold NMP, Acetone and IPA.

- SEM imaging of the JJ chain revealed the junction (or chain) width $\sim$ 510 nm, and the junction overlap $\sim$ 560 nm.

V. CURRENT-PEAK SPACINGS

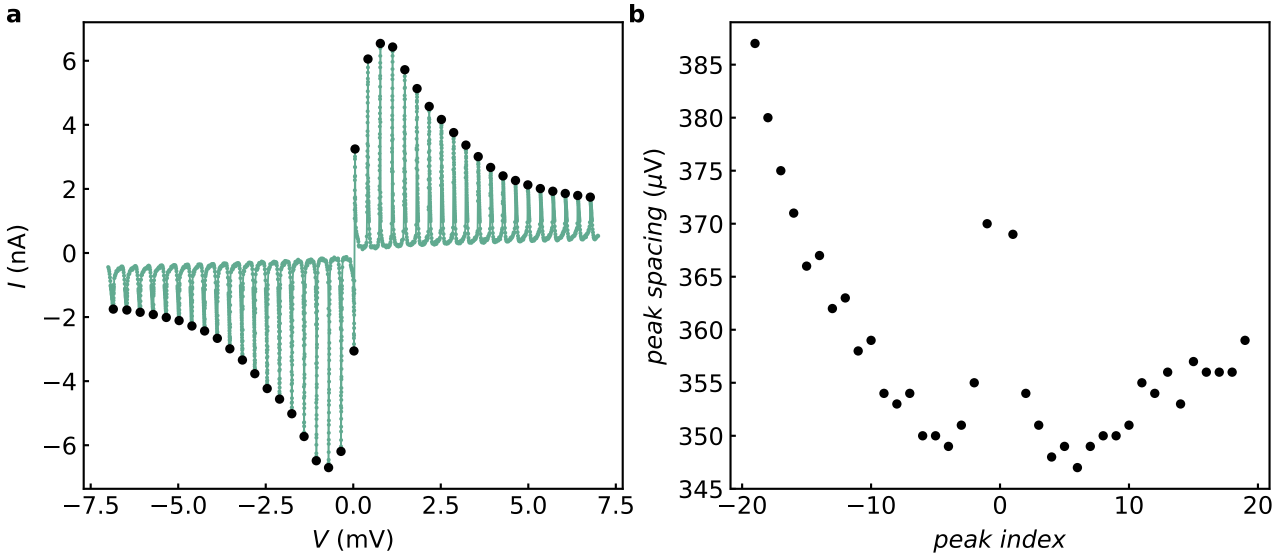

Figure S6. **Peak extraction and spacings.** **a,** Measured current I versus applied voltage V. The black dots represent detected current peaks. **b,** Peak spacings versus the peak index. Each peak index refer to the succeeding peak spacing on positive bias and to the preceding peak spacing on negative bias.

The typical peak spacing is comparable to twice the superconducting gap of Aluminum, 360 μeV. There is an overall smooth evolution of the peak spacings which is asymmetric in bias, which is not understood (Fig. S6b). Peak spacings are enhanced around zero bias, which we speculate is due to an interaction effect. Namely, the weakest link in the chain is the one which, due to offset charge disorder, is in the deepest Coulomb blockade. This link should switch first, and then require increased bias before current can flow. This picture predicts a that peak spacings should increase near zero bias, as we consistently observe.

VI. QUALITATIVE PICTURE OF FINITE-BIAS TRANSPORT

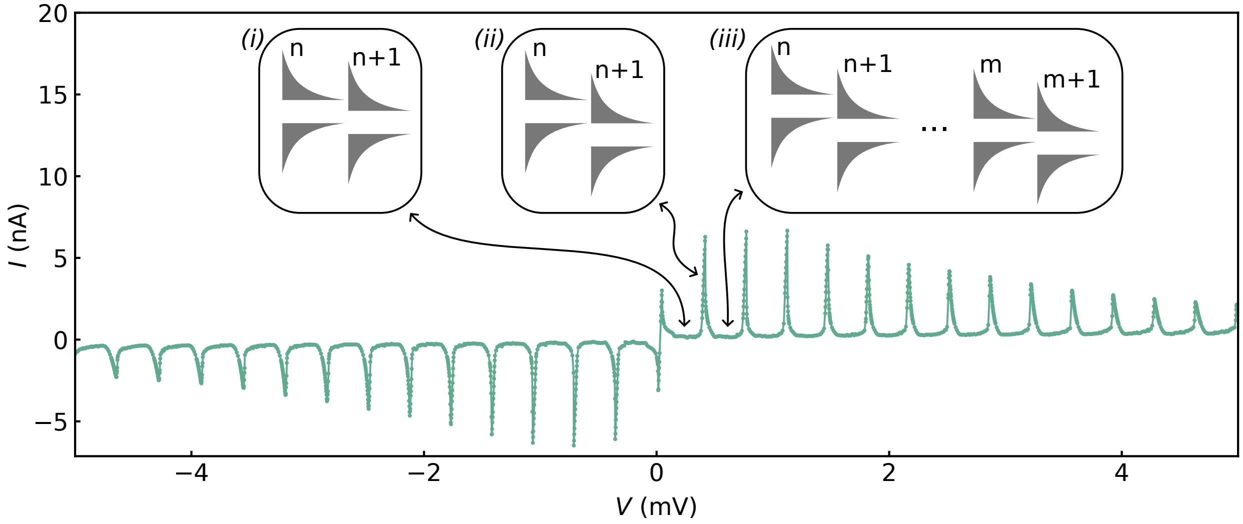

Figure S7. Current measured as a function of bias voltage at zero magnetic field, from Fig. 1c in the main text. Insets show schematic tunneling density of states of different islands at key voltages and positions along the chain. In configuration *(i)* the weakest junction, formed between islands n and $n+1$, has a voltage drop, with negligible current flowing due to the high resistance of the quasiparticle branch. In configuration *(ii)* a tunneling current flows through the weakest junction and a supercurrent flows through the other segments of the chain. In configuration *(iii)* the next-weakest junction, formed between islands m and $m+1$, is also in a voltage state, leading to negligible current similar to *(i)*, but at a voltage shifted by $2\Delta/e$.

Here we present a qualitative explanation of the approximately evenly spaced peaks observed in nonlinear transport. We argue that, although many of the transport processes are unclear, the basic phenomena can be understood as a series of switching events of weakly disordered Josephson junctions. Note that weak disorder is considered in Ref. [13] and does not alter the more detailed picture that we put forward for linear response in the main text. Similar observations and interpretations are found in Ref.'s [11, 20].

The sharp supercurrent branch at zero-bias is associated with a collective superconducting state of the entire array, with a resistance due to phase slips whose scaling with temperature and magnetic field are the focus of the main text. Current increases with bias until the critical current of the weakest junction is reached. At this point, a voltage drop develops over the weakest link, which effectively shuts off the current due to the high resistance of the quasiparticle branch [Fig. S7, inset (i)]. Once a bias out of 2Δ is reached, a tunneling current can flow through the junction, while a supercurrent is still supported in the rest of the chain [Fig. S7, inset (ii)]. As bias is further increased, the critical current of the next-weakest junction is reached, it goes into the quasiparticle branch, and the process is repeated [Fig. S7, inset (iii)]. This picture naturally explains a series of current peaks separated by voltage $2\Delta/e$. The initial increase in the height of current peaks can be understood as moving on to successively stronger junctions.

However, several of the observations lie completely beyond this picture. The decrease in the height of current peaks after the first few switches is unexpected, perhaps reflecting overheating of the chain. The smooth decrease in current after the first critical current is also unexpected, perhaps reflecting the contribution of a parallel shunting path from the electromagnetic environment. The differential conductance of the successive peaks is also not well understood (see Sec. VII).

VII. FINITE-BIAS DIFFERENTIAL CONDUCTANCE

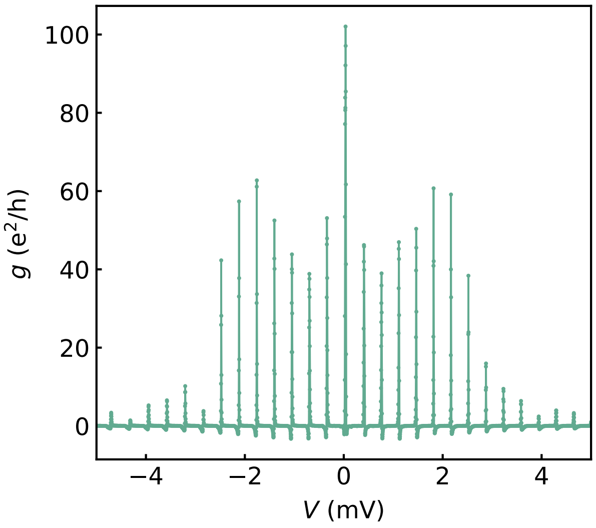

Figure S8. Measured differential conductance g versus applied voltage V, in a different cooldown. Data were measured in a two-terminal configuration with the same nominal line-resistance, 4.4 kΩ, subtracted as in the main text. Data are for zero magnetic field.

In a different cooldown of the same device, we studied finite-bias differential conductance in the superconducting state. A series of peaks in differential conductance are observed, consistent with the current-voltage characteristic presented in Fig. 1 in the main text. The conductance peak near zero-bias is largest, consistent with the identification of this as the collective supercurrent branch of the chain. Higher-bias peaks are smaller, but do not decrease monotonically as one would expect from the simple transport picture in Sec. VI, which is not understood.

VIII. BASE ELECTRON TEMPERATURE

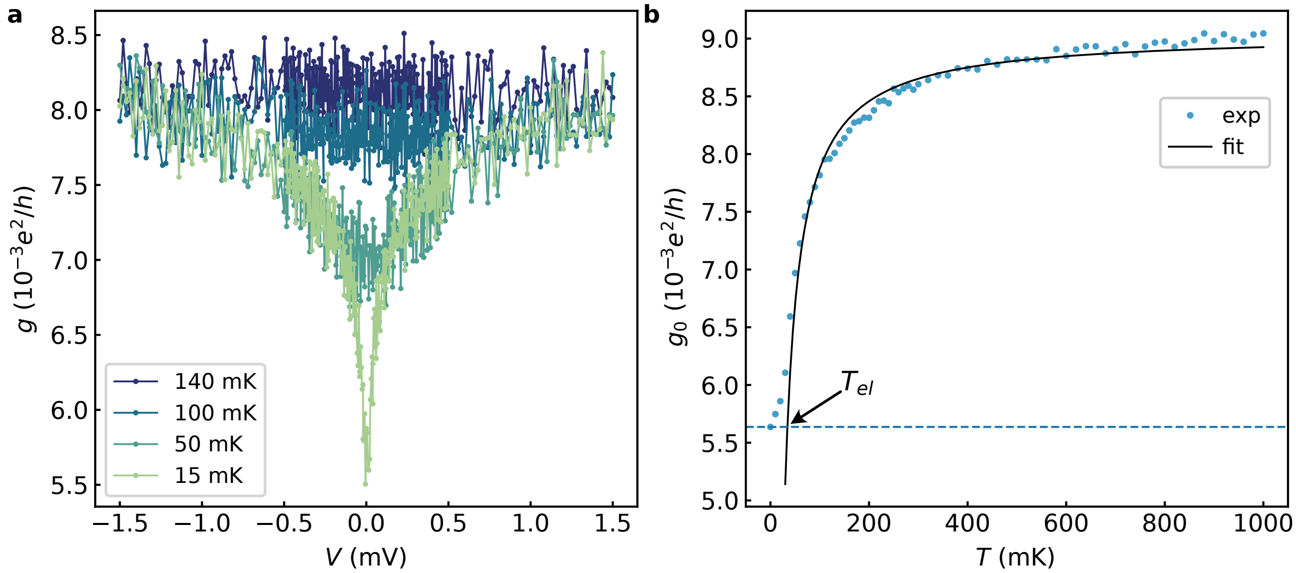

Figure S9. **Coulomb Blockade Thermometry at large magnetic fields. a,** Differential conductance g versus the applied voltage V, at various setpoint temperatures. **b,** Zero bias differential conductance g_0 versus the setpoint temperature T. Black line is a function fit to the data. The dashed blue line is the g_0 value corresponding to the lowest data point. T_{el} is the inferred base electron temperature of the system. Data in this figure is taken with the JJ chain being in normal state, at 500 mT.

Coulomb-blockade thermometry is performed in the normal state by applying a large, perpendicular magnetic field. Measuring differential conductance versus applied voltage shows a sharp dip at zero bias, which gets shallower on raising the temperature of the system (Fig. S9a). Extracting the zero bias differential conductance g_0 at all setpoint temperatures, shows a gradual decline in conductance below 400 mK, and a sharp fall below 100 mK (Fig. S9b). g_0 is fit to the well-known expression for Coulomb blockade thermometry [28, 44]

$$g_0 = g_T(1 - ((N-1)/N)E_C/(3k_BT)), \tag{S10}$$

where g_T is the asymptotic g at high bias voltages, N is the number of junctions in the chain and E_C is the charging energy of a junction. At high temperature the data agree well with Eq. S10. At low temperature the conductance is larger than the expected value, indicating that the device falls out of equilibrium with the cryostat. Associating the smallest observed conductance with a temperature gives the base electron temperature $T_{\mathrm{el}} = 35$ mK.

IX. COMPARING HIGH-BIAS TRANSPORT AND ZERO-BIAS PHASE DIAGRAM

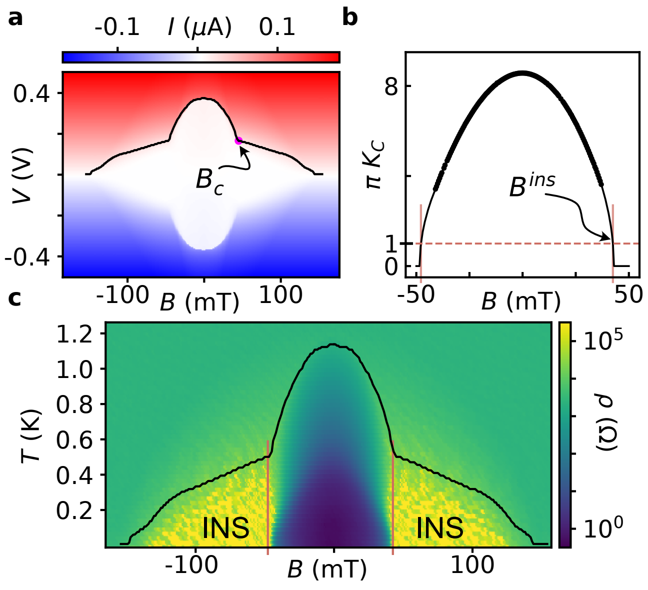

Figure S10. **Overlay of energy scales on the phase diagram.** **a,** Measured current I versus the bias voltage V and magnetic field B. The black curve indicates extracted critical voltage V_c. Kink in V_c at characteristic field B_c indicated. **b,** Inferred local superfluid phase stiffness πK_C versus the magnetic field B. Black dots are from experimental data. Solid black line is based on a linear extrapolation of $E_J(B)$ down to $E_J \to 0$, which is justified by the empirical observation that E_J is linear at high fields. B^{ins} is defined implicitly by $\pi K_C(B^{\text{ins}}) = 1$. **c,** The phase diagram with the zero bias differential specific resistance ρ as a function of the magnetic field B and temperature T. INS refers to Insulator. The black curve is the edge feature extracted from **a**, scaled by an empirical proportionality constant of $1/(N2\pi k_B)$. The vertical red lines in **b** and **c** correspond to the field values $|B| = B^{\text{ins}}$.

In the main text, we discussed that the wide-range current-voltage characteristic exhibits strongly suppressed current for biases below the critical voltage V_c. In this section we examine the field-dependence of V_c and point out that it resembles the high-temperature boundary in our experimental phase diagram. We also give further details on how we determine the expected value of B_{ins}.

Measuring current while varying bias voltage and magnetic field (Fig. S10a) reveals that V_c has a smooth field dependence up to a characteristic field value of $B_c = 45$ mT, at which point it exhibits a kink. After the kink V_c continues to decrease with magnetic field, eventually saturating at zero for high magnetic fields. The evolution of V_c empirically resembles the high-field boundary of the phase diagram, and B_c is comparable to B^{ins}.

The expected value of B^{ins} is found from the criteria $\pi K_C(B^{\text{ins}}) = 1$ (Fig. S10b), yielding a value $B^{\text{ins}} = 42.5$ mT at positive field. This matches the observed superconductor-insulator transition in Fig. 4b in the main text. Since B^{ins} lies slightly beyond the range where microwave measurements are possible, we have performed a linear extrapolation of $E_J(B)$ down to the regime $E_J \to 0$, justified by the linear behavior of E_J over a wide field range. B^{ins} is not perfectly symmetric in magnetic field, reflecting the fact that the field dependence of the microwave response is also not perfectly field-symmetric; this is not understood, but could indicate heating effects associated with the field sweep. Field-asymmetry is also present in the transport phase diagram, where the transition to the insulating state is also slightly field-asymmetric, and matches the asymmetry in B^{ins}. This suggests that the field asymmetry is a global and reproducible effect, consistent with heating.

X. MAGNETIC-FIELD TUNING

The purpose of this section is to give information on the expected magnetic-field evolution of the Josephson array. This discussion is relevant for understanding the limitations of the scaling theory at high magnetic field, and possible origins of the upper field boundary in the phase diagram. There are two important effects of the parallel magnetic field: a Fraunhofer-like suppression of Josephson energy, and a pair-breaking contribution. Precise understanding of the importance of these effects would require knowledge of the magnetic field distribution in both the junction and island regions, which is a formidable task. We offer some simple estimates of the importance of these effects below.

description	value (mT)	origin
first Fraunhofer critical field	65 ± 10	scaled from wider device
thin island critical field	220 ± 20	Ref. [45]
thick island critical field	70 ± 20	Ref. [45]

Table SV. Summary of expected critical fields.

The first important effect is the suppression of the Josephson energy due to the threading of magnetic flux into an effective area defined by the barrier magnetic thickness and the width of our array [46]. In wider, lower-impedance arrays we have observed clear revivals of supercurrent features, which we interpret as Fraunhofer interference due to this effect. Scaling for the thinner width of the arrays we study here, we estimate an expected first Fraunhofer critical field reported in Table. SV.

The second important effect is the suppression of the superconducting gap due to pair-breaking effects. A simple point of comparison are the known thin-film critical magnetic field of Al tabulated in Ref. [45], which we have used to extract expected critical fields for our two island thicknesses in Table. SV.

Comparing the Fraunhofer and pair-breaking field scales, a few observations can be made. The first Fraunhofer critical field and the thick island critical field are comparable. This indicates that the superconductor-insulator transition we observe, which is at a slightly smaller field, could involve not just phase fluctuations, but amplitude fluctuations and quasiparticles. The agreement between theory and experiment in Fig.'s 3-4 in the main text, even near the SIT, suggests that amplitude fluctuations and quasiparticles do not dominate the physics at low temperatures. However, they could well explain the quantitative deviations from power-laws we observe at higher temperatures and magnetic fields.

It is also interesting to note that the expected critical field of the thin islands is substantially larger than the high-field boundary of our phase diagram. As mentioned in the main text, after the high-field boundary, the temperature dependence of the array resistance is consistent with the normal state (shown in Sec. VIII). This discrepancy could possibly be due to flux-focusing effects or a slight misalignment of the chip plane with the magnetic field.

XI. MEASUREMENT AND ANALYSIS CHECKS

In this section we discuss some of the checks that we have performed to minimize systematic errors in our measurement and analysis procedures.

A. Lock-in excitation

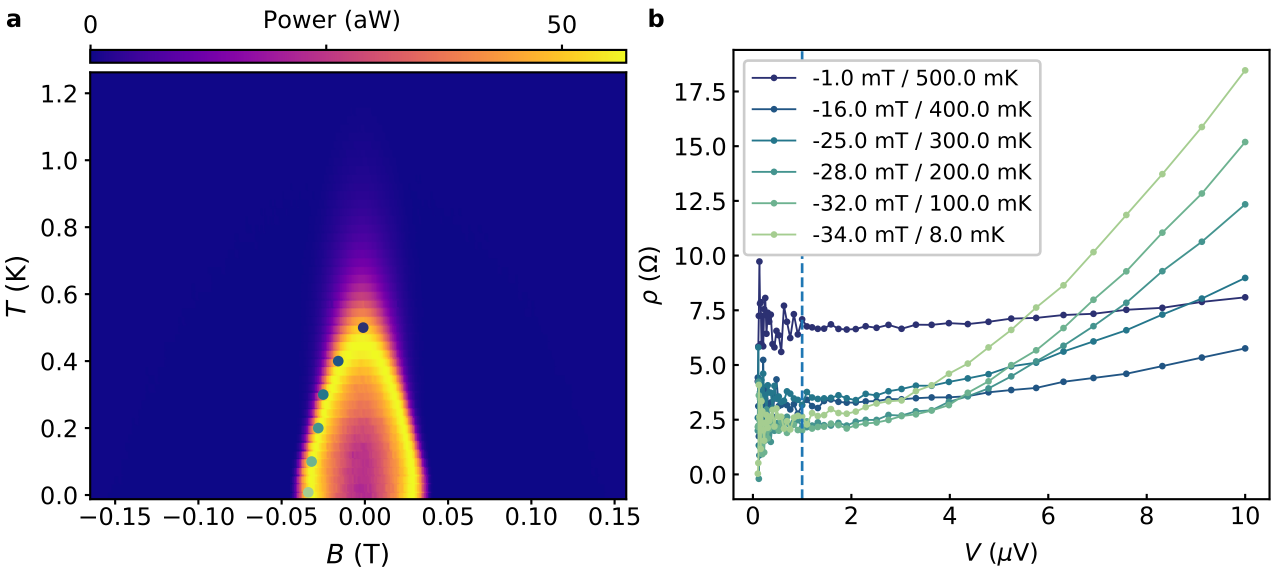

Figure S11. **Heating check for the transport JJ chain.** **a,** Power dissipated at the chain versus temperature T and magnetic field B. **b,** Zero bias differential specific resistance ρ versus lock-in voltage amplitude V: at various (B, T) points along the region of maximal power dissipation in **a**. The dashed line indicates the voltage (1 μV) used for measuring the phase diagram. The temperatures indicate the setpoint temperature of the cryostat.

During experiments we recorded both the two-probe and four-probe zero bias differential resistance. Throughout the main text, the two-probe resistance is plotted with the inferred line resistance from a four-probe measurement subtracted. This procedure removes technical noise at the expense of introducing a small (few Ohm) systematic error in the data. A Stanford SR830 was used to measure the differential current dI through the chain, before which the current signal was converted to voltage using Basel LSK389A transimpedance amplifier. The differential voltage drop across the chain, dV was measured with a Zurich MFLI. A voltage amplitude of 1 μV was applied, which we experimentally verified was sufficiently small to avoid overheating.

Mapping out power dissipated ($dI \times dV$) at the device over full (B,T) parameter space (Fig. S11a), reveals a dome-like feature of maximal power dissipation. Choosing a few points over the dome to do an amplitude study (Fig. S11b), reveals that the device lies comfortably in the linear response regime for our chosen excitation of 1 μV.

B. Higher lock-in excitation for Fig. 4b

To resolve $\rho(T)$ curves near the superconductor-insulator transition, as in Fig. 4b in the main text, we found it useful to adjust our lock-in settings. In this regime the device resistance is large, which results in small measured currents at fixed excitation voltage, but fortunately also allows for larger excitations without encountering Joule heating. We made two changes:

1. Turning up the voltage amplitude to 10 μV.

2. Measuring the dI, dV over ten time points and recording the mean value.

To check for Joule heating due to the increased lock-in excitation, we performed an amplitude study similar to the one depicted in Fig. S11b.

C. Lower-field resistance upturn

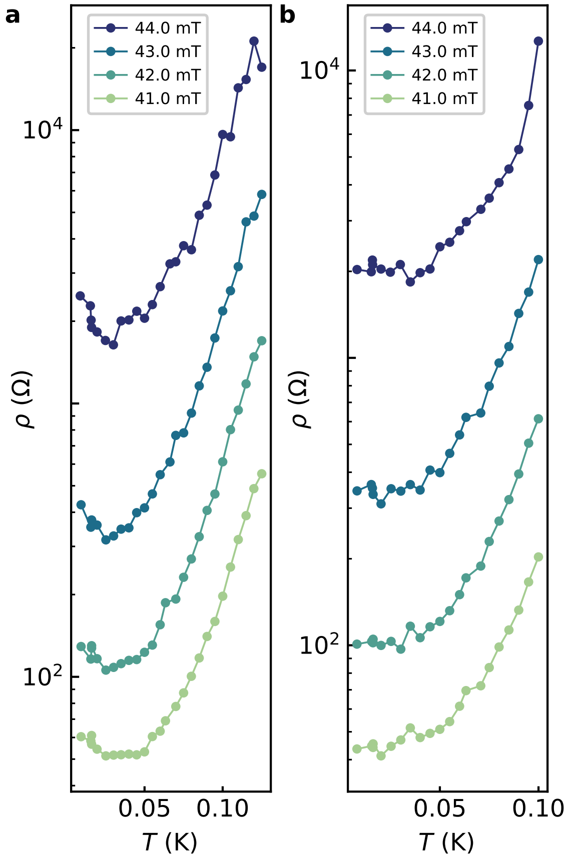

Figure S12. **Lower-field resistance upturn. a,** Zero-bias differential specific resistance ρ as a function of measured temperature T, at various magnetic fields. **b,** The same measurement repeated on a different run and same cooldown.

As pointed out in Fig. S12, a low-temperature upturn in specific resistance is inconsistently observed at fields a few mT before the reproducible SIT that we identified in the main text. We interpret the intermittently-observed low-temperature upturns as a signature of us being on the edge of resolving the crossover to insulating behavior, which should always occur for sufficiently low temperature. This is consistent with our picture that T^* is renormalized upwards as the system enters strong coupling for $\pi K_C \sim 1$.

D. Voltage Offsets

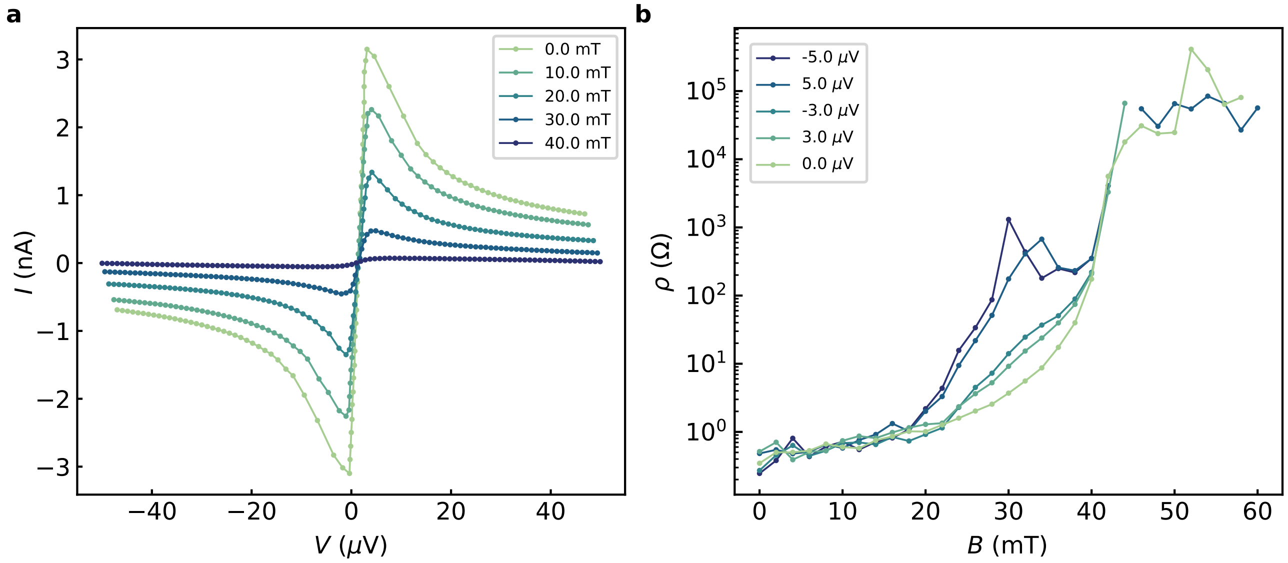

Figure S13. **Effect of voltage offsets in specific resistance measurement. a,** Current I versus the applied voltage V, in the narrow bias range, measured at various magnetic fields. **b,** Zero bias differential specific resistance ρ versus the magnetic field B, with and without applied offset voltages.

The supercurrent peak, and the zero bias conductance, gradually decreases on increasing the magnetic field (Fig. S13a). Lock-in setup measures the slope of the zero bias peak. As shown in Fig. S13b, adding voltage offsets to the lock-in measurement changes the behavior of the device in the region where the device transitions from LSC to INS phase (refer to Fig. 4c in main text). Hence, ensuring proper offset correction at zero voltage bias is essential to measurement of the phase diagram.

E. Power law fits

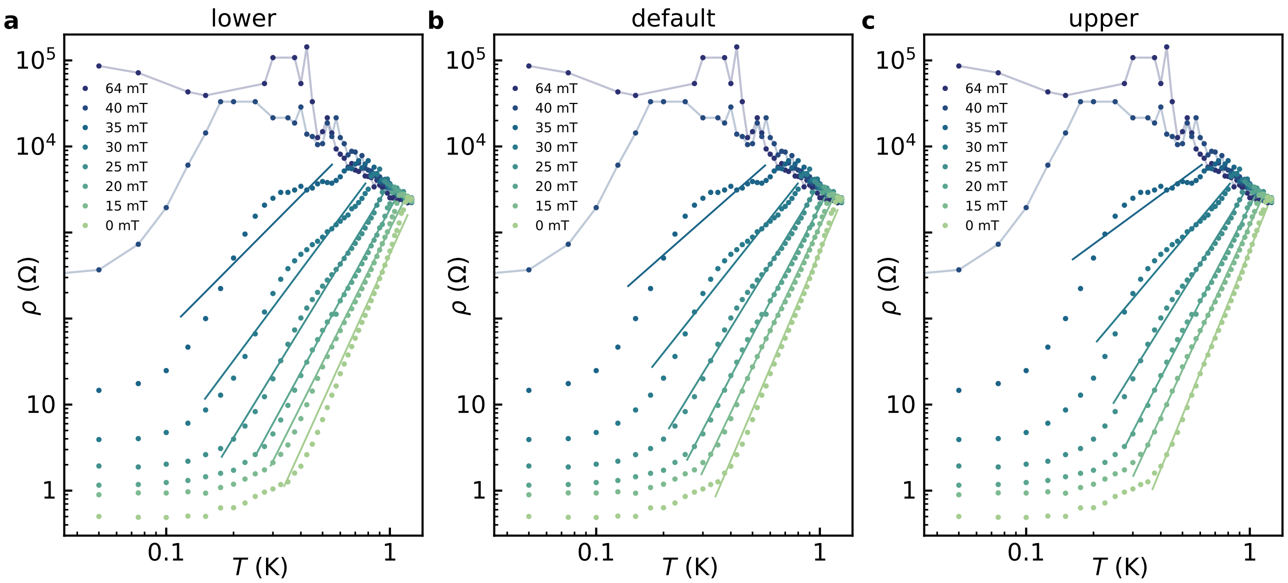

Figure S14. **Power law fits with various choices of lower cutoffs.** **a,** Zero bias differential specific resistance ρ versus temperature T, with 0.18 $\mathrm{T_P}$ as the lower cutoff temperature for power law fits. **b,** Same plot with 0.215 $\mathrm{T_P}$ as the lower cutoff temperature for fits. **c,** Same plot with 0.25 $\mathrm{T_P}$ as the lower cutoff temperature for fits. $\mathrm{T_P}$ is the plasma frequency in temperature units.

To analyze the power-law behavior of $\rho(T)$, fits must be performed over a restricted temperature range. Because system parameters evolve with magnetic field, the fit range must also be field dependent. The high-temperature limit of the fitting range is chosen to be 95% of the plasma temperature T_P, where

$$T_{\mathrm{p}} = \sqrt{2E_J(B)E_C}/k_B. \tag{S11}$$

As shown in the main text, the upper edge of the local superconducting dome follows the plasma temperature, so this is a suitable upper bound.

The low-temperature limit of the fitting range is not as easy to sharply define, due to the smooth crossover to saturating specific resistance. To account for this difficulty, we explored a range of lower cutoff values, as shown in Fig. S14. These cutoff values are used to create the blue error bands for the exponent p in Fig. 3b of the main text. To evaluate the impact of the systematic error bands on fit parameters, $p(K_c)$ was fit to a line for the three lower cutoffs in Fig. S14. The range of values obtained are reported as uncertainties in the slope and intercept in the main text.

F. Comparison of experiment with infrared theory

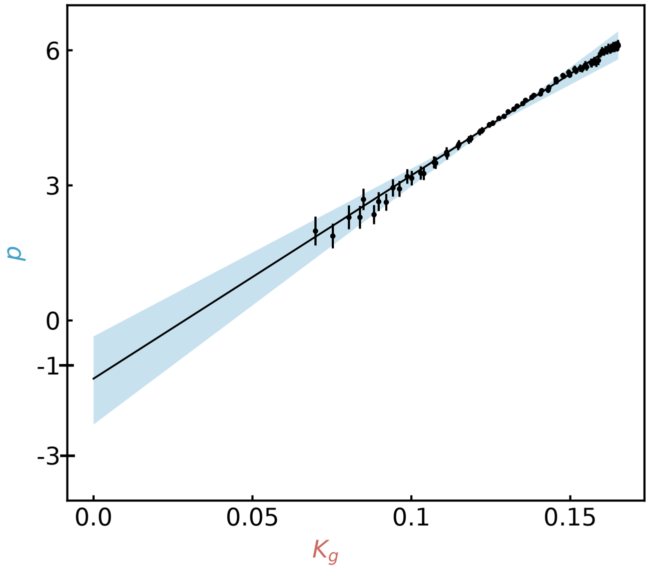

Figure S15. **Comparing fit exponents with global superfluid phase stiffness.** Exponent p from power-law fits versus the global superfluid phase stiffness K_g from microwave measurements. Solid line is a linear fit. Shaded blue region depicts range of linear fits obtained by repeating the entire analysis with different low-temperature cutoffs for the power-law fits (Sec. XI E). Center of the shaded region is a low-temperature cutoff $0.215T_P$, which was used throughout the analysis. Error bars are standard error from the power law fits.

Solving the Renormalization Group (RG) equations in the UV limit, yields a power law with exponent $\pi K_C - 1$ (Eq. S17), where K_C is the bare value of K in RG flow. While solving in the IR limit, with renormalized K, results in a power law with exponent $2\pi K_g - 3$.

Comparing p from the transport measurements with the global superfluid phase stiffness K_g inferred from microwave measurements reveals a linear behavior (Fig. S15) with slope 45 ± 7 and intercept of -1.3 ± 1.0. This in complete disagreement with the predicted slope of 2π for global superconductivity. The intercept close to -1 is same as observed for local superconductivity (Fig. 3b in main text), owing to the fact that only the x-axis is scaled down on plotting versus K_g.

G. Power-law extraction of crossover temperature

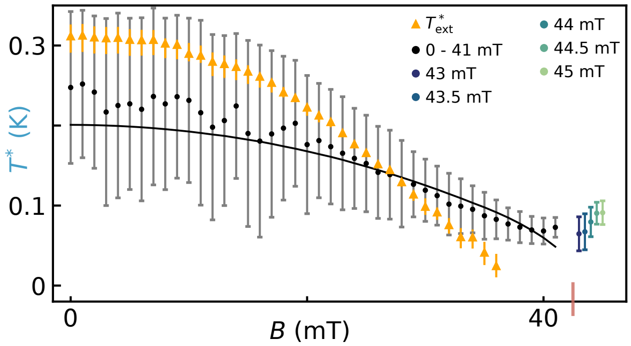

Figure S16. **Power-law extraction of crossover temperature.** Crossover temperature extracted using power-law extrapolation ($T^*_{\rm ext}$, orange triangles), which is accurate only at low magnetic field, compared with method from the main text (markers, identical from Fig. 4a), as a function of applied magnetic field. T^* and it's error bars are identical to those in Fig. 4a; Vertical error bars derived from the percentage above minimum resistance (5%, 150%) for the (lower,upper) ranges. Error bars in $T^*_{\rm ext}$ determined from the different low-temperature fit cutoffs in Sec. XI E, the (lower,upper) limits are from ($0.18T_P$, $0.25T_P$), and central value is the center of this range, $0.215T_P$. See Fig. S14 for examples of the fits with each cutoff. Solid line is theoretical prediction, also identical to Fig. 4a in the main text.

In this section we provide a check that our metric T^* from the main text reasonably identifies observed saturation temperatures by comparing it with an alternate metric based on extrapolating the power law fits. In the main text, a characteristic saturation temperature T^* is identified based on the fractional change in resistance above its minimum value. We chose this metric because it performs well both with curves having crossovers to saturation and with those having minima, without making reference to any theoretical assumptions. Since the definition is not physically motivated, we included generous error bars in Fig. 4a.

To check if the error bars are reasonable, we compare our metric with another sensible definition based on the power-law behavior of the data. When the power-law behavior is quantitatively very well satisfied, which only holds at modest magnetic fields, the fit power-law can be used to find an extrapolated saturation temperature $T^*_{\rm ext}$ according to

$$T^*_{\rm ext} = (R_{\rm min}/A)^{1/p}, \tag{S12}$$

where $R_{\rm min}$ is the minimum resistance of the $R(T)$ curve, A is the fit power-law amplitude, and p is the fit power-law exponent. Fig. S16 compares $T^*_{\rm ext}$ with the metric used for T^* from the main text over the full range of data. Up until the highest magnetic fields, where the data begin to deviate from a power law, T^* and $T^*_{\rm ext}$ agree, indicating that the error bars are representative of the uncertainty in T^*.

H. Low wave-number resonant frequencies

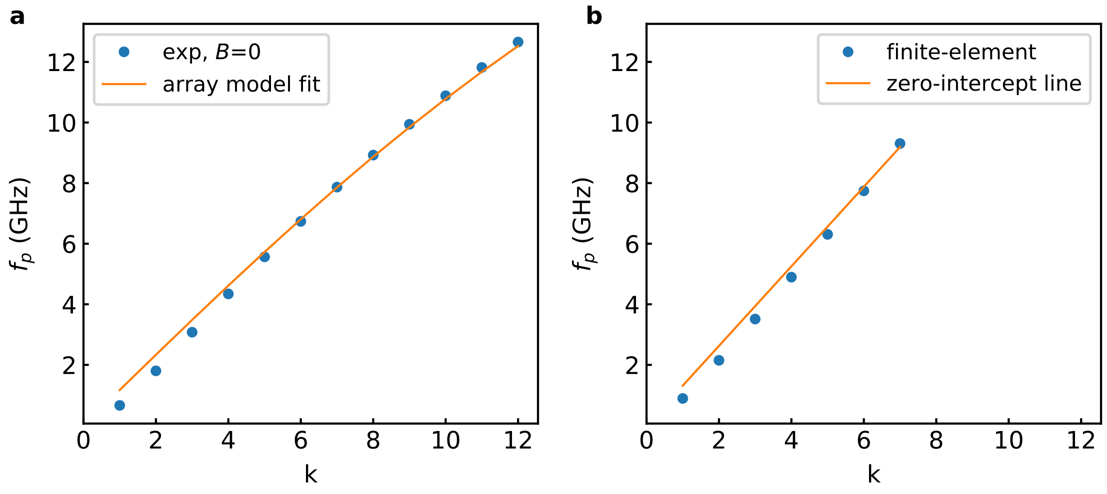

Figure S17. **Comparison of data and electromagnetic simulation.** Resonant frequencies for the Josephson array at $B = 0$ with fit dispersion relation (**a**), and for a simulated device (**b**). Zero-intercept line is fit to the three highest-k points. The simulation is for a zero-resistance, high-inductance metal, which is equivalent to an ideal Josephson array with $\omega_p \to \infty$.

The observed dependence of plasma mode frequencies on wavenumber agrees with Eq. S2 reasonably well, as shown in Fig. S1b. However, the first few resonant frequencies are lower than theoretically expected. To check if this is associated with the electromagnetic environment of our device, we have performed finite-element simulations of a high-impedance transmission line resonator with identical coupling capacitors and ground-plane configurations, and an inductance per unit length adjusted to give approximately the same fundamental resonant frequency as our measured device. In this configuration, one naively expects evenly spaced modes with resonant frequencies satisfying $f_p \propto n$ where n is the mode number. This can be thought of as a Josephson array with $\omega_p \to \infty$. In other words, the electromagnetic simulation can only capture the acoustic part of the Josephson array dispersion.

In the electromagnetic simulation, resonant frequencies do not lie along a line as naively expected. Rather, low mode-number points tend to fall below a zero-intercept line, which is reminiscent of the behavior of low mode-number points in the real device [Fig. S17]. Our interpretation is that the low-wavenumber modes in the acoustic part of the Josephson array spectrum have reduced frequencies due to an electromagnetic effect associated with our chip layout.

XII. ADDITIONAL ANALYSIS

A. Scaling

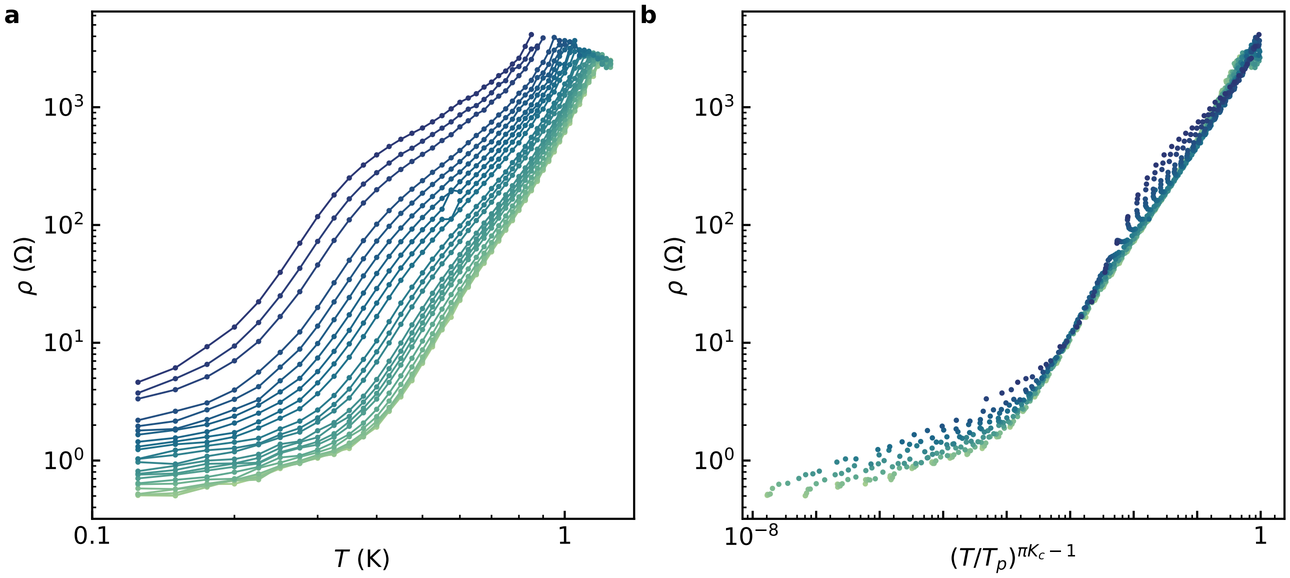

Figure S18. **Collapse of power laws. a,** Zero bias differential specific resistance ρ as a function of temperature T, at various magnetic fields. **b,** The same data in **a** plotted with scaled temperature axis, where T_P is the plasma frequency in temperature units and K_C is the local superfluid phase stiffness.

As shown in Fig. S18a, the power law behavior of specific resistance with temperature is observed until about 40 mT, albeit with shoulder-like features emerging at high field. In Fig. S18b, scaling the normalized temperature axis with expected exponent collapses all the data on left into an universal power law behavior, removing the field dependency of the data. As pointed out earlier in Eq. S11, T_P is a field dependent quantity.

B. Planckian limit

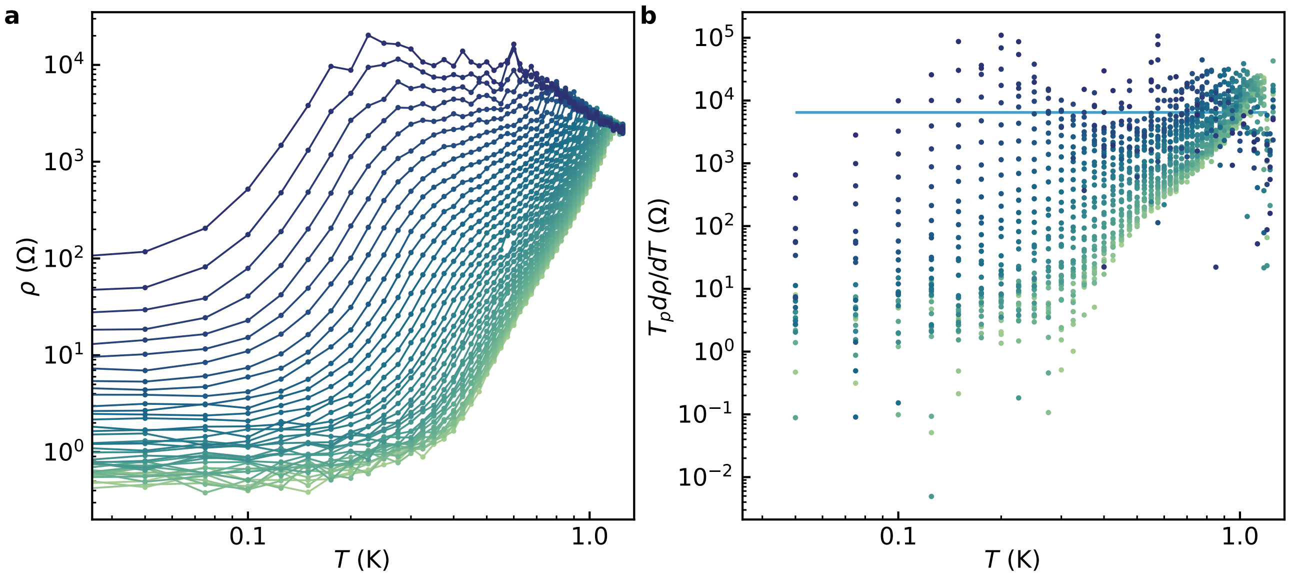

Figure S19. **Planckian slope check. a,** Zero bias differential specific resistance ρ as a function of temperature T, at various magnetic fields. **b,** The slope of the curves in **a**, times the plasma frequency $\mathrm{T_P}$, versus temperature T. The blue horizontal line is the resistance quantum, R_Q.

Our picture for local superconductivity connects the timescale of thermal fluctuations near quantum criticality, $\tau = h/(k_B T)$, to specific resistance. In the literature there is a widely hypothesized connection between the Planckian scattering time $\tau_s = h/(k_B T)$ and quantum-critical metals [47–51]. Given recent reports of Planckian scattering in a superconductor-insulator system [37], and of the qualitative similarity of some of our specific resistance curves to Ref. [37], we were motivated to directly compare our data with a model of Planckian scattering. Following the method of Ref. [37], we computed $T_P d\rho/dT$, where the plasma temperature T_P plays the role of the high-temperature cutoff for superconducting behavior in our system. The Planckian bound is $T_P \cdot d\rho/dT < h/(4e^2)$. As shown in Fig. S19b, the observed specific resistance exceeds this bound by more than an order of magnitude. Thus, the Planckian bound apparently does not apply in our system.

XIII. COMPARISON WITH PREVIOUS WORK

Pioneering transport measurements identified a superconductor-insulator transition in sufficiently long arrays [10, 11]. In light of our current understanding, and the availability of theories making the correct phase stiffness expressions unambiguous [13], it is interesting to revisit this work. As discussed below, we find that our current understanding accounts for the observations of Ref. [10] in sufficiently long arrays. In shorter arrays, as pointed out in Ref. [10], finite-size effects are dominant.

Estimating system parameters from [10], we find $\pi K_c \sim 5.5$ and $\pi K_g \sim 0.5$ at zero magnetic field. Our picture therefore predicts a zero-temperature insulator but a high-temperature superconductor. The authors observe that $R(T)$ decreases with lowering T, which we interpret as superconducting behavior, and saturates at low T, which we interpret as a crossover to the insulating regime. The saturation crossover temperature T^* initially decreases with increasing magnetic field, then begins increasing after the observed SIT. All of these observations are compatible with our picture, and similar to the observations in Fig. 4a,4b in the main text. Indeed, we have estimated that their observed SIT occurs at $\pi K_c \sim 1$. A remarkable difference between our data and Ref. [10] is that we observe clear power-law scaling at low magnetic fields. This difference is most likely explained by the fact that our devices are approximately an order of magnitude longer, which allows the thermal length to serve as the limiting scale over a broader parameter range.

XIV. OUTLOOK FOR ACCESSING INFRARED PHYSICS

We have presented evidence that high-temperature effects dramatically alter the physics of the superconductor-insulator transition in one-dimensional Josephson arrays. It would be appealing to access the long-sought infrared superconductor-insulator physics, corresponding to a Giamarchi-Schulz transition in the dirty limit or Berezinskii–Kosterlitz–Thouless (BKT) in the clean limit [3, 13, 27]. In terms of our phase diagram, the infrared limit superconductor-insulator limit (IR-SIT) is indicated in Fig. S20.

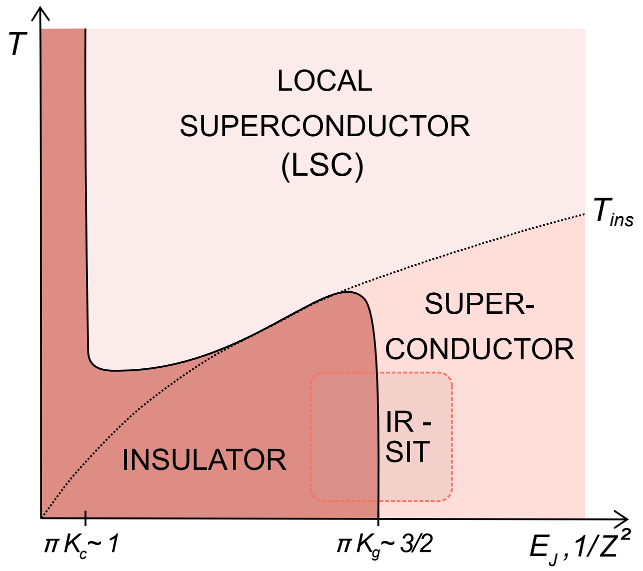

Figure S20. **Infrared limit.** Proposed phase diagram with infrared superconductor-insulator (IR-SIT) region indicated.

The challenge of observing this region in experiments is pushing up $T_{\rm ins}$ to higher temperatures. In our opinion the most straightforward way of doing this is to decrease the charge-screening length $\Lambda = \sqrt{E_g/E_c}$, which could be accomplished with the minimal change of increasing the capacitance to ground. This will also naturally move samples towards the IR-SIT region, which is beneficial since currently they are expected to be deep in the insulating regime at zero temperature. We have estimated that $T_{\rm ins} \sim 150$ mK and $\pi K_g \sim 3/2$ is experimentally achievable with this approach. This value of $T_{\rm ins}$ is much larger than typical dilution-refrigerator base temperatures (10 mK), which indicates that the IR-SIT may be accessible. An important problem to address experimentally is to what extent systems can be thermalized near the IR-SIT.

XV. THEORY

Here we give an overview of the theoretical origin of power-law scaling discussed in the main text.

The key simplification for our case arises from the fact that the dimensionless phase slip rate,

$$y \propto e^{-4\sqrt{2E_J/E_C}} \tag{S13}$$

is small, less than 10^{-20} at zero magnetic field. We note that different pre-factors sometimes appear in the literature inside the exponential in Eq. S13. Our expression is identical to Refs. [23–26]. Ref. [13] calculated a larger pre-factor in the infinite screening length limit and discussed its origin. The exact value of the pre-factor does not affect our results.

The renormalization group equations from Ref. [13], linearized for small y and in the long-screening-length limit

$(\Lambda \gg 1)$ are

$$\begin{aligned} \frac{dK}{dl} &= -K(1-u_g) && \text{(S14)} \\ \frac{du_g}{dl} &= 2u_g(1-u_g) && \text{(S15)} \\ \frac{dy}{dl} &= \frac{1+u_g}{2}(2-\pi K)y. && \text{(S16)} \end{aligned}$$

Here K is the superfluid phase stiffness, taking the initial value $K_C = \sqrt{E_J/2E_C}$ and u_g takes the initial value $1/(1+\Lambda^2)$, where Λ is the charge screening length, representing the plasmon group velocity in the UV limit in units of the plasma frequency. Following Ref. [13], we assume the renormalization flow is terminated at the thermal length given by $e^l = \Omega_\mathrm{p}/T$ where $\Omega_\mathrm{p} = \sqrt{2E_JE_C}$ is the single junction plasma frequency. The resistance is then given by $R = R_0 y^2/e^l$.

Equations (S14-S15) express the renormalization of K from its ultraviolet value of K_C down to K_g at the fixed point $u_g = 1$.

In the high temperature limit where K is hardly renormalized, resistance follows the power law behavior

$$R = R_0 \left(\frac{T}{\Omega_\mathrm{p}}\right)^{\pi K_C - 1}. \tag{S17}$$

At lower temperature K is renormalized down and the system crosses over to insulating behavior. The crossover temperature depends on system parameters,

$$T_\mathrm{ins} = \max(2E_C/\pi, \sqrt{2E_JE_C}/\Lambda). \tag{S18}$$

The first case occurs in the limit of small Josephson energy, and insulating behavior appears because K is renormalized below $1/\pi$, at which point the system enters strong coupling. The second case occurs in the limit of large Josephson energy, and insulating behavior appears because the system crosses over to the infrared limit where $u_g = 1$, which is the case we focus on in the main text.

In the experiment, the two terms in Eq. S18 are actually comparable. A more complete formula for T_ins would need to incorporate both of these effects, but, at the level of accuracy at which T_ins can be experimentally identified, the formula in the main text is sufficient.

A. Boundaries in theoretical phase diagram

The superconductor-insulator transition is strictly only a phase transition at $T = 0$; otherwise it is a crossover [2]. The theoretical phase diagram in the main text in fact labels crossover boundaries, where the temperature dependence of the specific resistance $d\rho/dT$ changes sign. To identify these points, we work perturbatively in the limit of small phase-slip rate, as discussed above.

At low temperatures where the infrared fixed-point of Eqs. (S14-S15) is reached, the $\rho(T)$ power law is $2\pi K_g - 3$, which gives the low-temperature crossover $\pi K_g \sim 3/2$. Note that once terms of order y^2 are included into Eq. S16, one would actually find either the Giamarchi-Schulz or BKT fixed points depending on if disorder is included [2, 27]. Since this correction is small compared to those associated with local superconductivity discussed in the main text, we simply indicate the crossover point with a $\sim$ to avoid ambiguity.

By similar logic, in the high-temperature limit $\rho(T)$ power law is $\pi K_C - 1$, which yields the local superconductor-insulator crossover $\pi K_C \sim 1$.

The boundary between local and global regimes is given by Eq. S18, which when smoothly interpolated yields the theoretical diagram in the main text.
