# Supplementary figures and images for: Superconductivity from a melted insulator in Josephson junction arrays
Source: Nat Phys. 2023 Aug 10;19(11):1630–5. doi: 10.1038/s41567-023-02161-w (PMC10635826; doi:10.1038/s41567-023-02161-w)

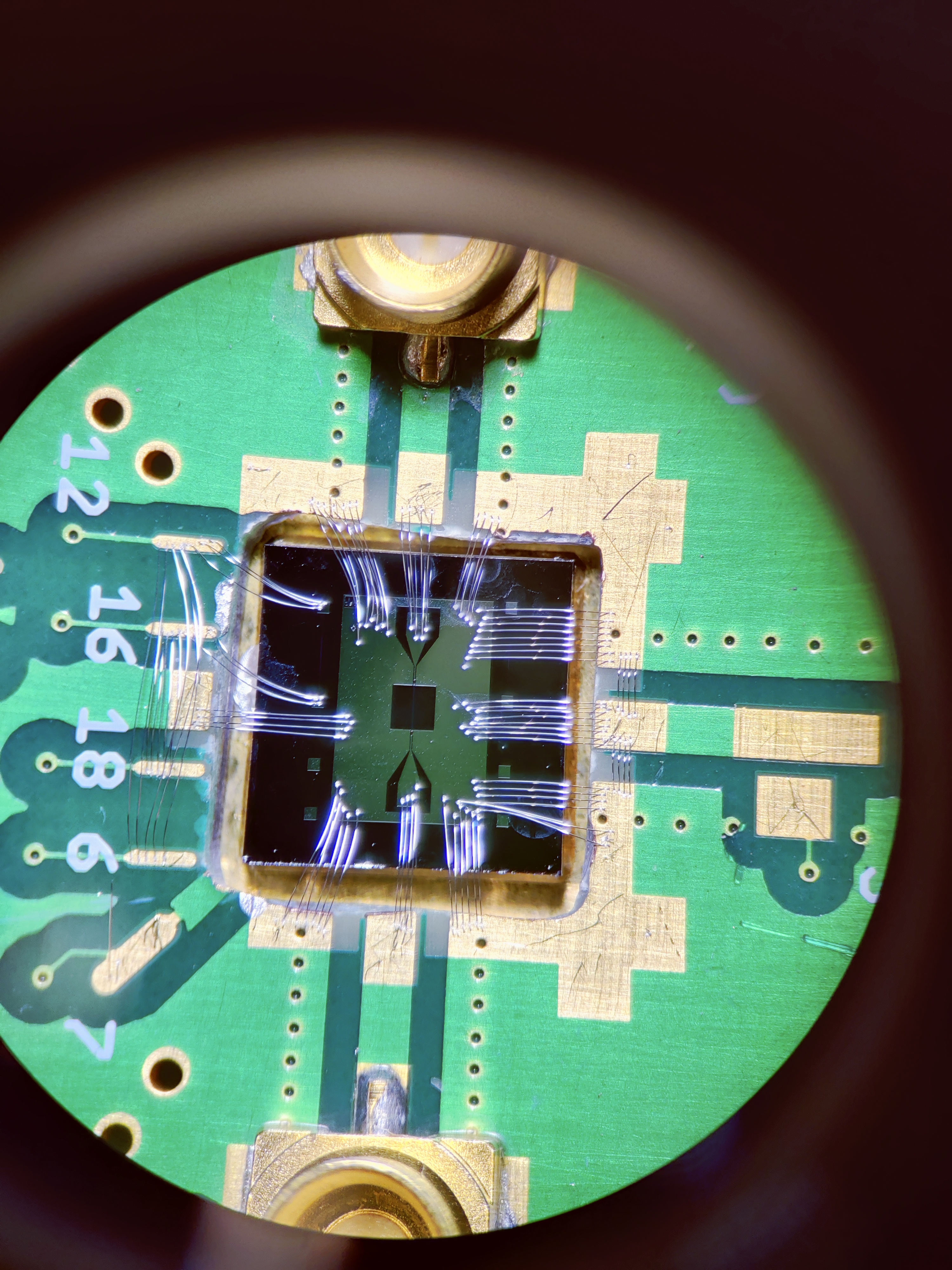

Supplement: Supplementary file 2 — Contains all data in the paper, including supplement, serialized into text files, and Python scripts that recreate all figures from the text files. [file 41567_2023_2161_MOESM2_ESM.zip › data_and_plotters_all/phase_figures/supfigs/f9s2_bonded.jpg]

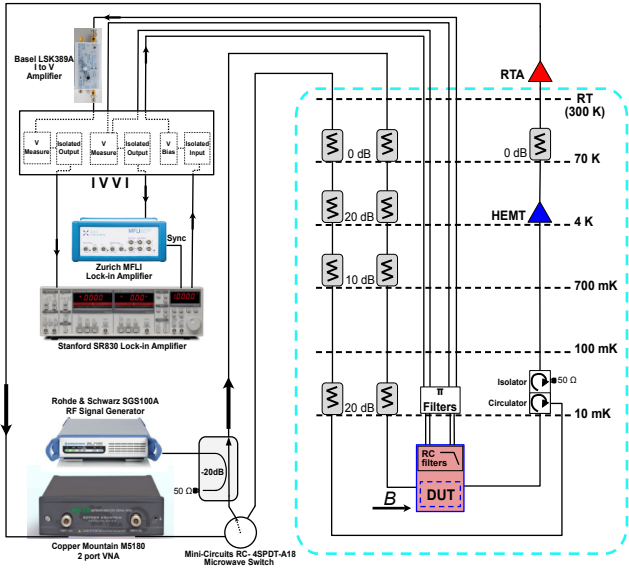

Supplement: Supplementary file 2 — Contains all data in the paper, including supplement, serialized into text files, and Python scripts that recreate all figures from the text files. [file 41567_2023_2161_MOESM2_ESM.zip › data_and_plotters_all/phase_figures/supfigs/experimental_setup.pdf]

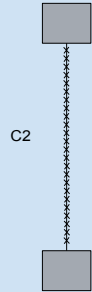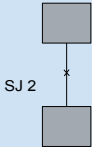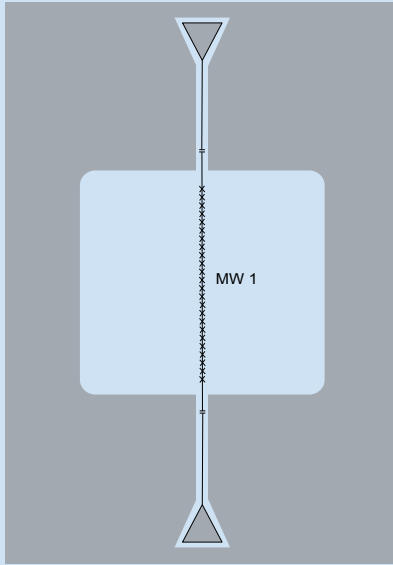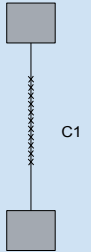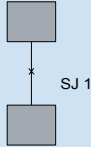

Supplement: Supplementary file 2 — Contains all data in the paper, including supplement, serialized into text files, and Python scripts that recreate all figures from the text files. [file 41567_2023_2161_MOESM2_ESM.zip › data_and_plotters_all/phase_figures/supfigs/chip_schematic.pdf]

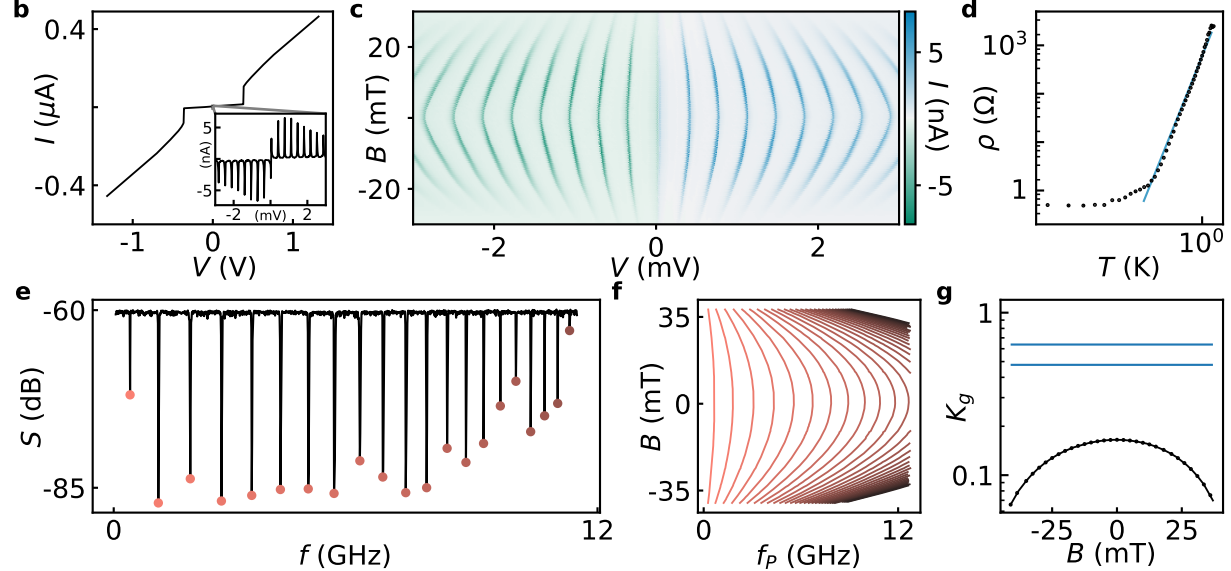

Supplement: Supplementary file 2 — Contains all data in the paper, including supplement, serialized into text files, and Python scripts that recreate all figures from the text files. [file 41567_2023_2161_MOESM2_ESM.zip › data_and_plotters_all/phase_figures/fig1/Fig1.pdf]

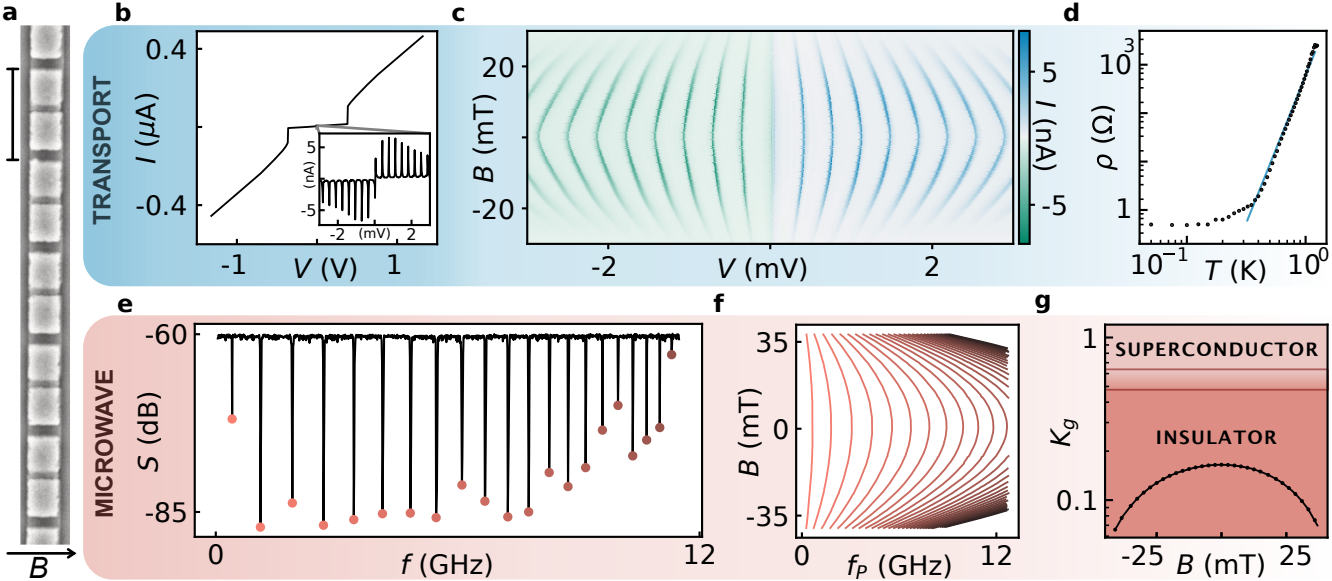

Supplement: Supplementary file 2 — Contains all data in the paper, including supplement, serialized into text files, and Python scripts that recreate all figures from the text files. [file 41567_2023_2161_MOESM2_ESM.zip › data_and_plotters_all/phase_figures/fig1/Fig1_design.pdf]

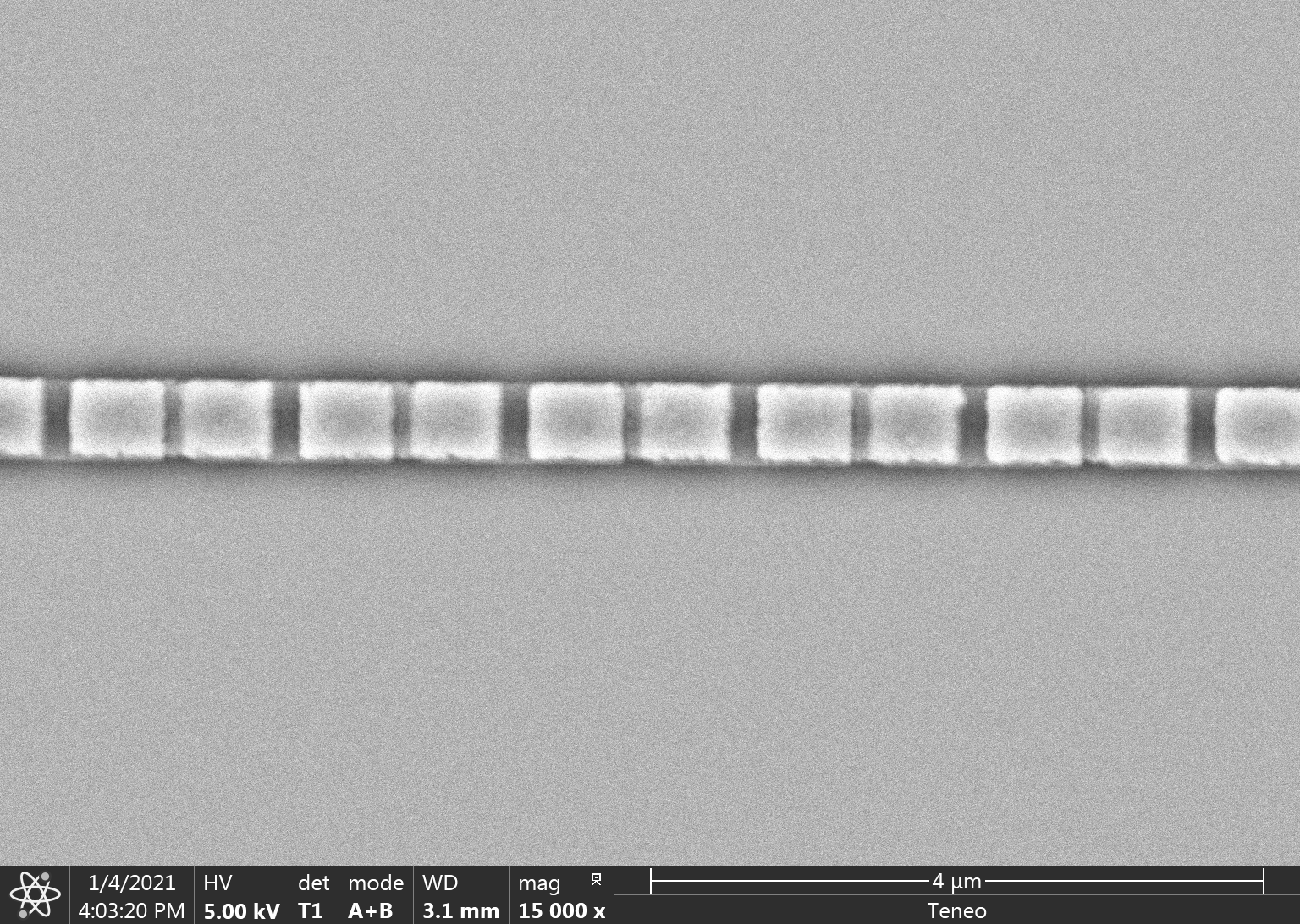

Supplement: Supplementary file 2 — Contains all data in the paper, including supplement, serialized into text files, and Python scripts that recreate all figures from the text files. [file 41567_2023_2161_MOESM2_ESM.zip › data_and_plotters_all/phase_figures/fig1/mw _chain_500_nm.tif]

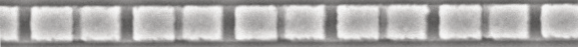

Supplement: Supplementary file 2 — Contains all data in the paper, including supplement, serialized into text files, and Python scripts that recreate all figures from the text files. [file 41567_2023_2161_MOESM2_ESM.zip › data_and_plotters_all/phase_figures/fig1/jj_chain.pdf]

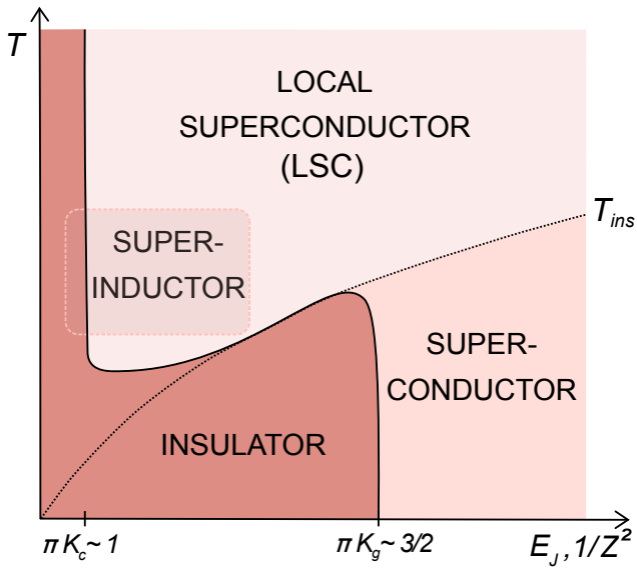

Supplement: Supplementary file 2 — Contains all data in the paper, including supplement, serialized into text files, and Python scripts that recreate all figures from the text files. [file 41567_2023_2161_MOESM2_ESM.zip › data_and_plotters_all/phase_figures/fig2/Fig2_design.pdf]

**a**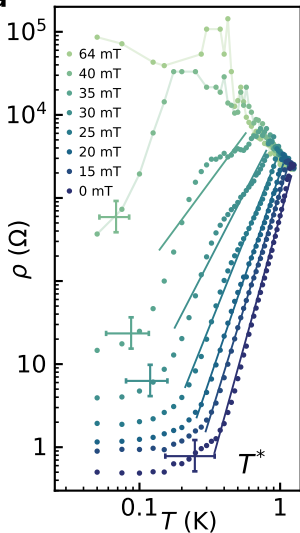**b**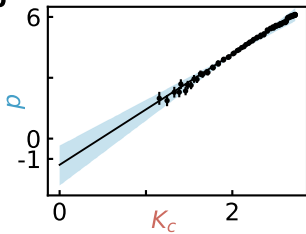**c**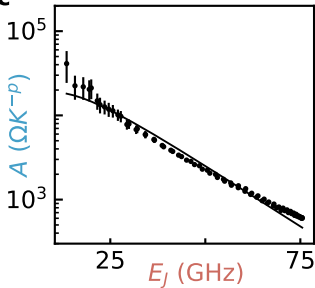

Supplement: Supplementary file 2 — Contains all data in the paper, including supplement, serialized into text files, and Python scripts that recreate all figures from the text files. [file 41567_2023_2161_MOESM2_ESM.zip › data_and_plotters_all/phase_figures/fig3/Fig3.pdf]

**a**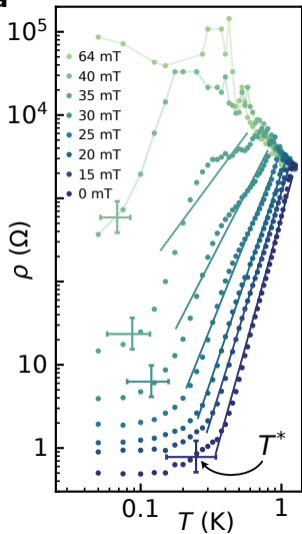**b**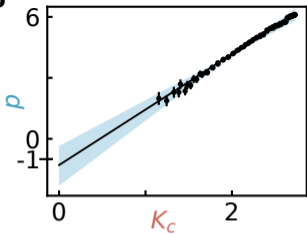**c**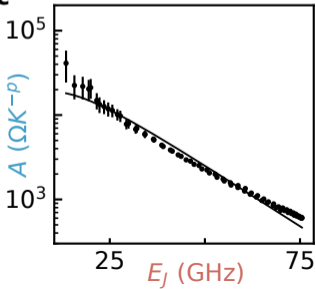

Supplement: Supplementary file 2 — Contains all data in the paper, including supplement, serialized into text files, and Python scripts that recreate all figures from the text files. [file 41567_2023_2161_MOESM2_ESM.zip › data_and_plotters_all/phase_figures/fig3/Fig3_design.pdf]

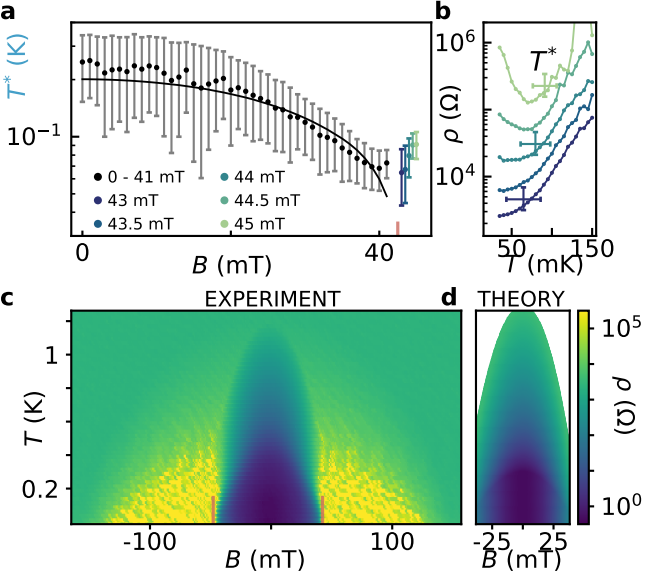

Supplement: Supplementary file 2 — Contains all data in the paper, including supplement, serialized into text files, and Python scripts that recreate all figures from the text files. [file 41567_2023_2161_MOESM2_ESM.zip › data_and_plotters_all/phase_figures/fig4/Fig4.pdf]

**a**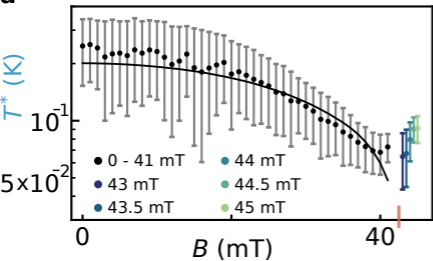**b**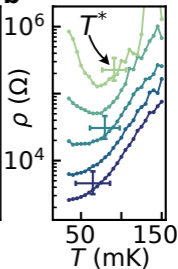**c**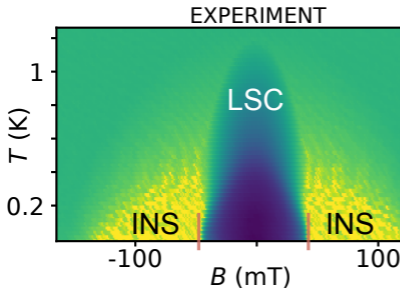**d**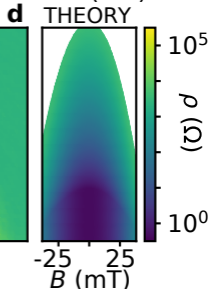

Supplement: Supplementary file 2 — Contains all data in the paper, including supplement, serialized into text files, and Python scripts that recreate all figures from the text files. [file 41567_2023_2161_MOESM2_ESM.zip › data_and_plotters_all/phase_figures/fig4/Fig4_design.pdf]

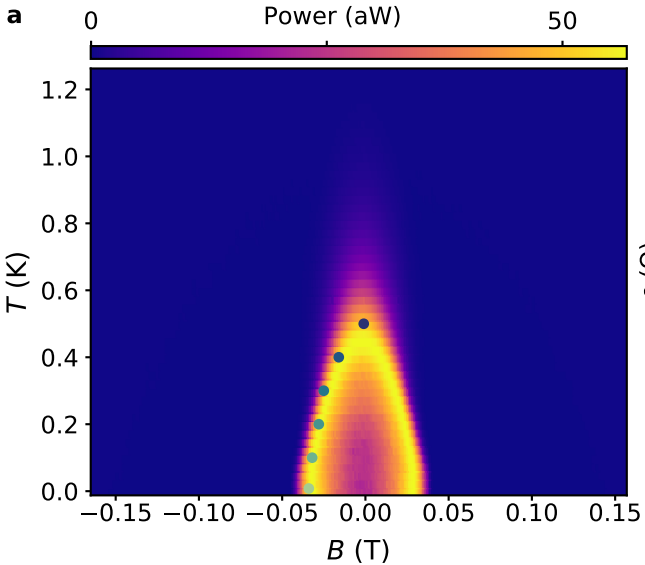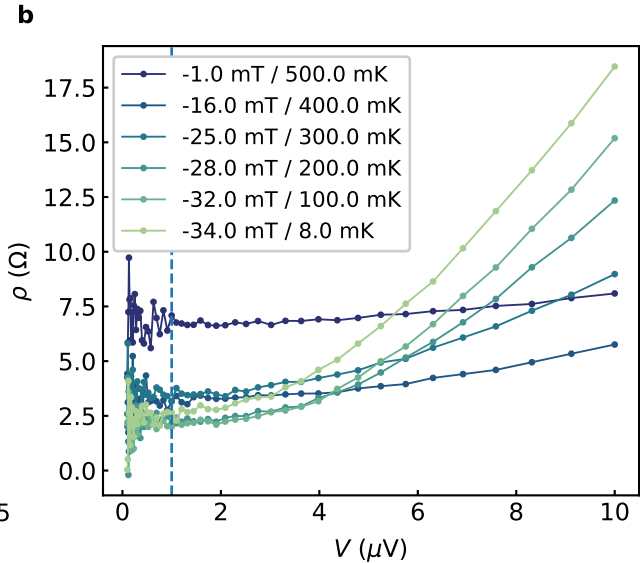

Supplement: Supplementary file 2 — Contains all data in the paper, including supplement, serialized into text files, and Python scripts that recreate all figures from the text files. [file 41567_2023_2161_MOESM2_ESM.zip › data_and_plotters_all/phase_figures/supfigs/sfig_heatmap/sfig_heatmap.pdf]

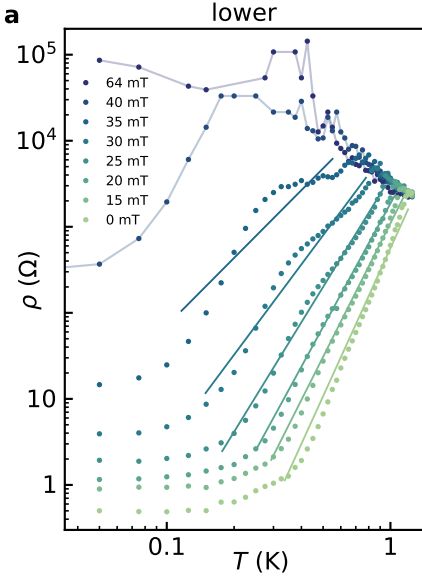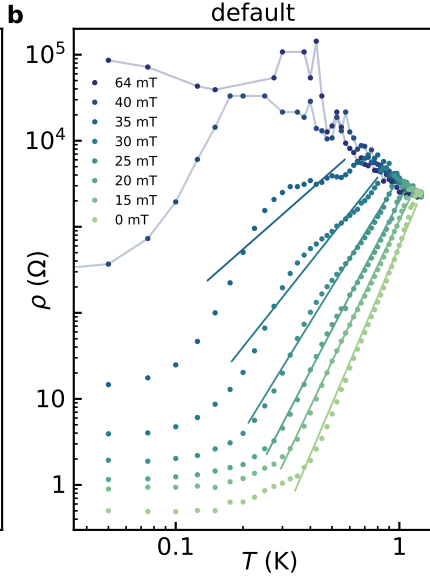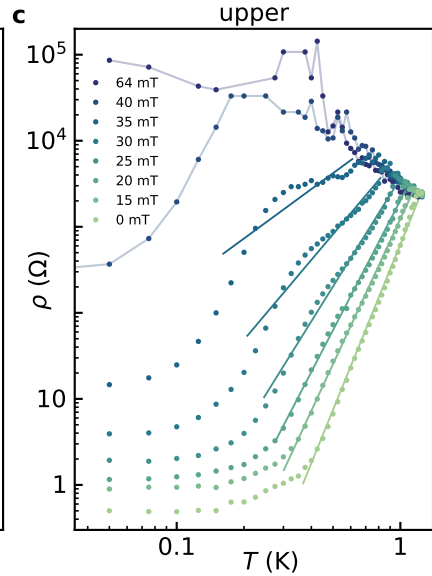

Supplement: Supplementary file 2 — Contains all data in the paper, including supplement, serialized into text files, and Python scripts that recreate all figures from the text files. [file 41567_2023_2161_MOESM2_ESM.zip › data_and_plotters_all/phase_figures/supfigs/sfig_plaw_fits/sfig_plaw_fits.pdf]

**a**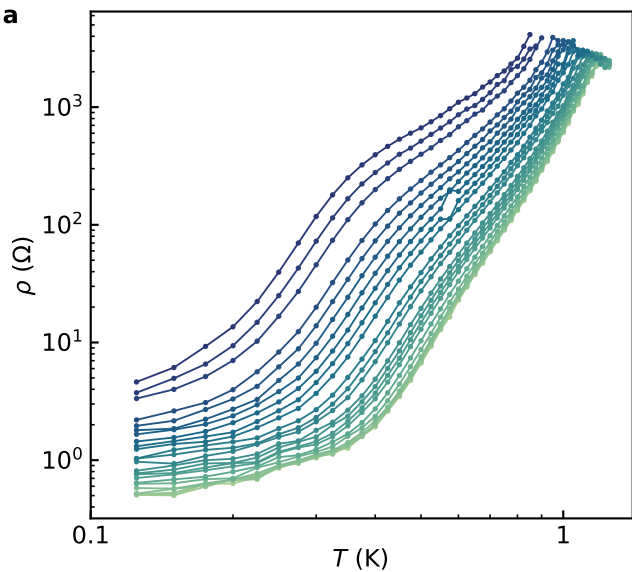**b**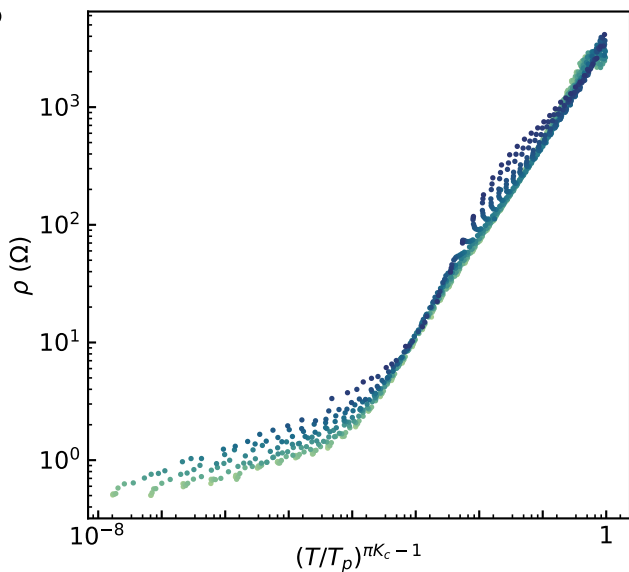

Supplement: Supplementary file 2 — Contains all data in the paper, including supplement, serialized into text files, and Python scripts that recreate all figures from the text files. [file 41567_2023_2161_MOESM2_ESM.zip › data_and_plotters_all/phase_figures/supfigs/sfig_collapse/sfig_collapse.pdf]

**a**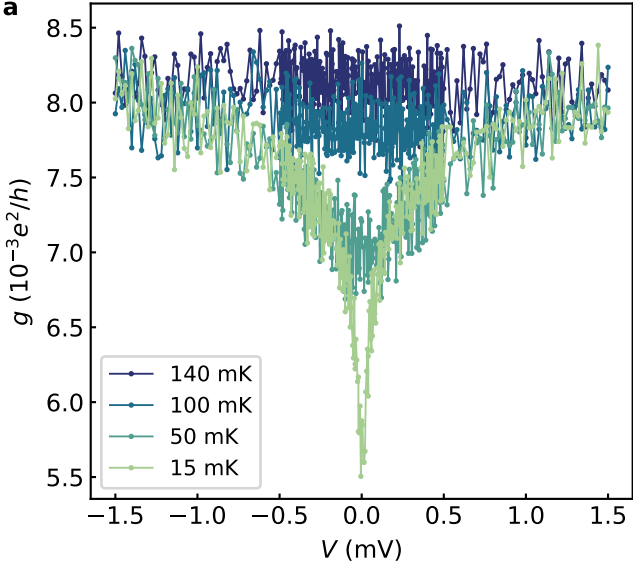**b**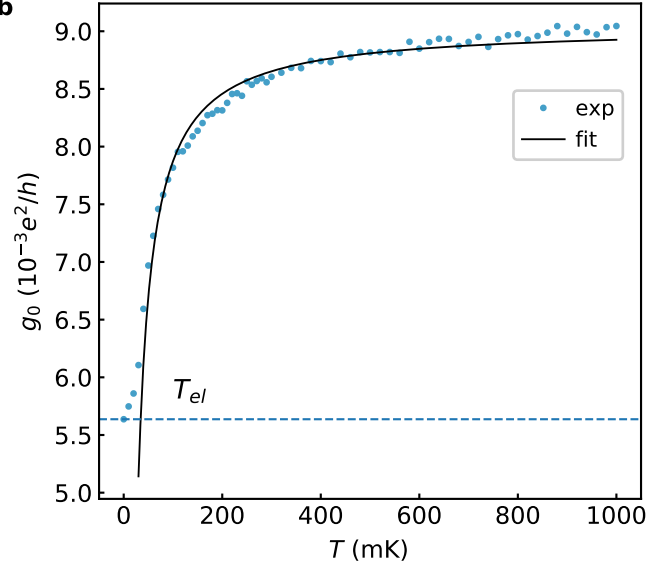

Supplement: Supplementary file 2 — Contains all data in the paper, including supplement, serialized into text files, and Python scripts that recreate all figures from the text files. [file 41567_2023_2161_MOESM2_ESM.zip › data_and_plotters_all/phase_figures/supfigs/sfig_cbt/sfig_cbt.pdf]

**a**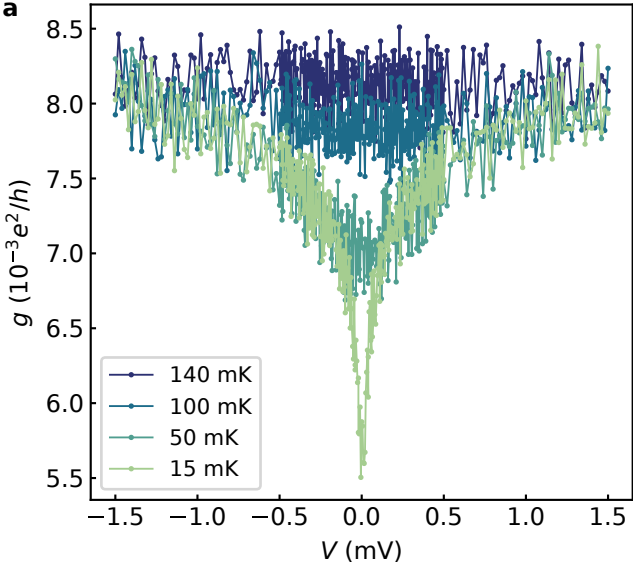**b**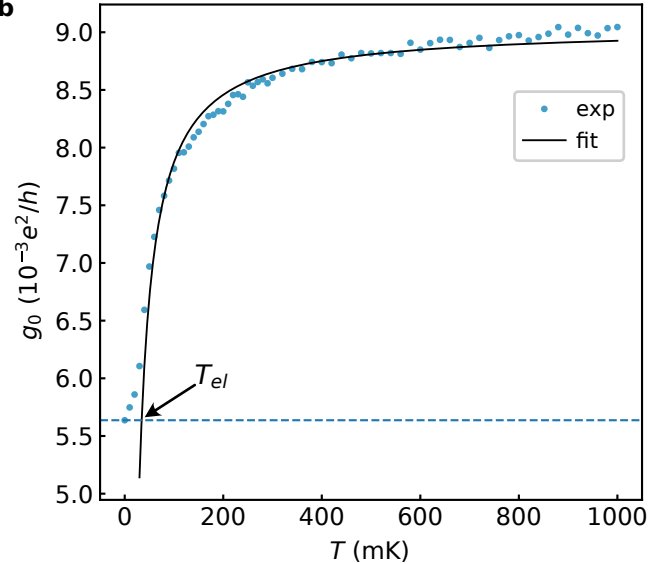

Supplement: Supplementary file 2 — Contains all data in the paper, including supplement, serialized into text files, and Python scripts that recreate all figures from the text files. [file 41567_2023_2161_MOESM2_ESM.zip › data_and_plotters_all/phase_figures/supfigs/sfig_cbt/sfig_cbt_design.pdf]

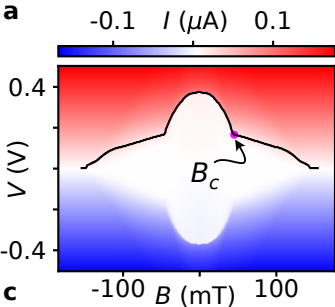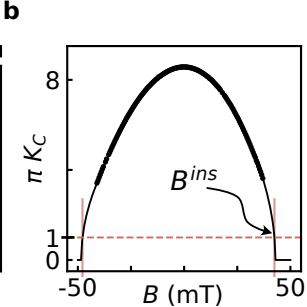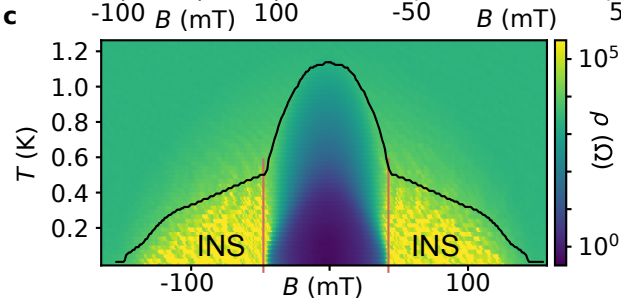

Supplement: Supplementary file 2 — Contains all data in the paper, including supplement, serialized into text files, and Python scripts that recreate all figures from the text files. [file 41567_2023_2161_MOESM2_ESM.zip › data_and_plotters_all/phase_figures/supfigs/sfig_bc_bins/sfig_bc_bins_design.pdf]

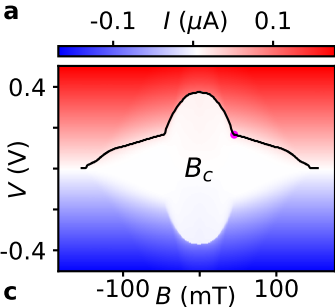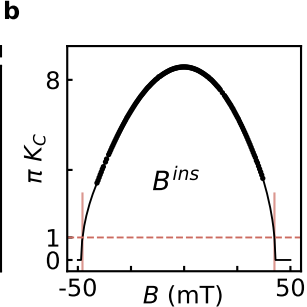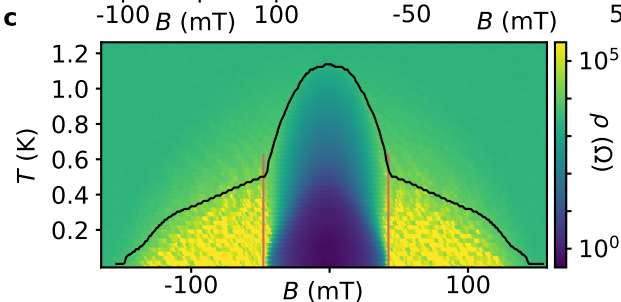

Supplement: Supplementary file 2 — Contains all data in the paper, including supplement, serialized into text files, and Python scripts that recreate all figures from the text files. [file 41567_2023_2161_MOESM2_ESM.zip › data_and_plotters_all/phase_figures/supfigs/sfig_bc_bins/sfig_bc_bins_.pdf]

**a**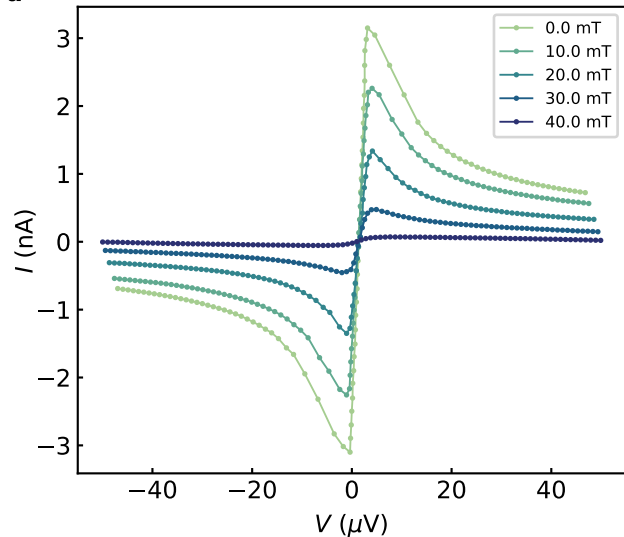**b**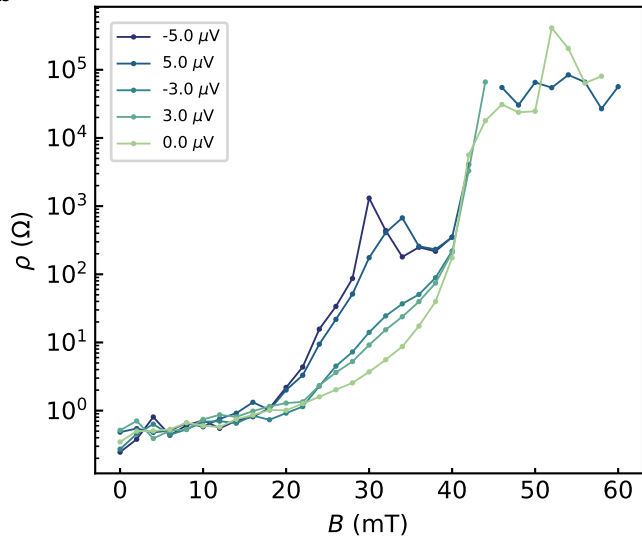

Supplement: Supplementary file 2 — Contains all data in the paper, including supplement, serialized into text files, and Python scripts that recreate all figures from the text files. [file 41567_2023_2161_MOESM2_ESM.zip › data_and_plotters_all/phase_figures/supfigs/sfig_v_offset/sfig_v_offset.pdf]

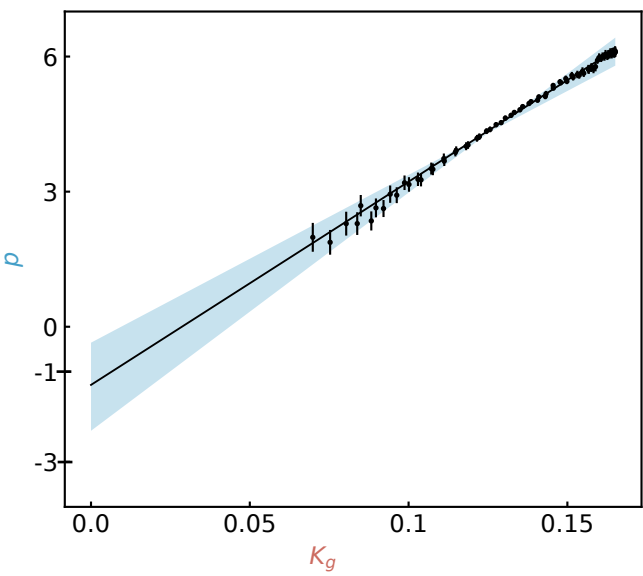

Supplement: Supplementary file 2 — Contains all data in the paper, including supplement, serialized into text files, and Python scripts that recreate all figures from the text files. [file 41567_2023_2161_MOESM2_ESM.zip › data_and_plotters_all/phase_figures/supfigs/sfig_kg/sfig_kg_design.pdf]

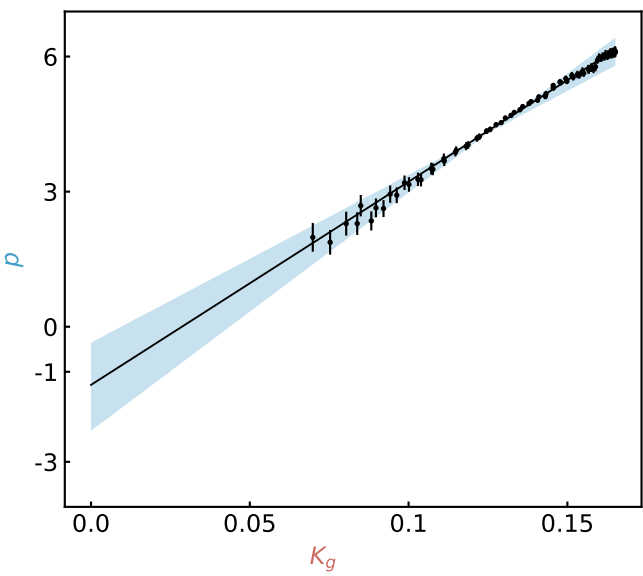

Supplement: Supplementary file 2 — Contains all data in the paper, including supplement, serialized into text files, and Python scripts that recreate all figures from the text files. [file 41567_2023_2161_MOESM2_ESM.zip › data_and_plotters_all/phase_figures/supfigs/sfig_kg/sfig_kg.pdf]

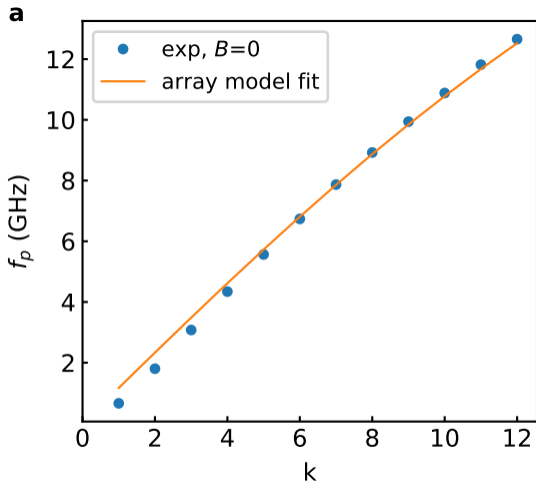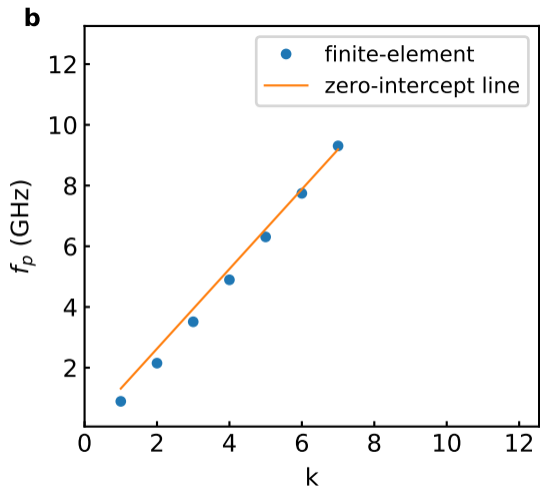

Supplement: Supplementary file 2 — Contains all data in the paper, including supplement, serialized into text files, and Python scripts that recreate all figures from the text files. [file 41567_2023_2161_MOESM2_ESM.zip › data_and_plotters_all/phase_figures/supfigs/sfig_em_disper/sfig_em_disper.pdf]

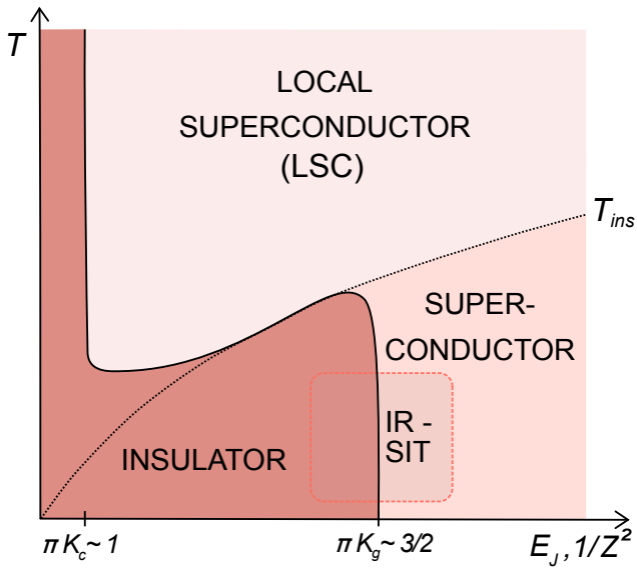

Supplement: Supplementary file 2 — Contains all data in the paper, including supplement, serialized into text files, and Python scripts that recreate all figures from the text files. [file 41567_2023_2161_MOESM2_ESM.zip › data_and_plotters_all/phase_figures/supfigs/sfig_ir_sit/sfig_ir_sit_design.pdf]

**a**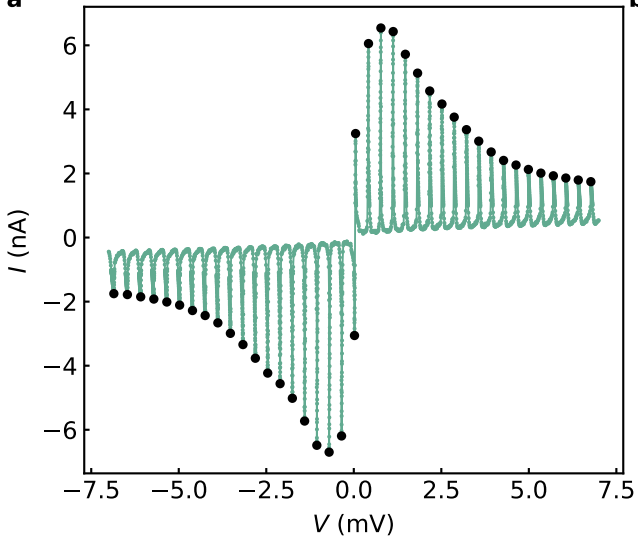**b**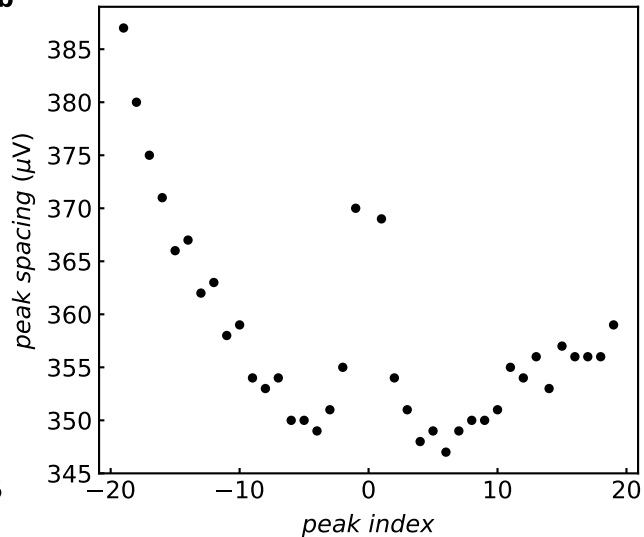

Supplement: Supplementary file 2 — Contains all data in the paper, including supplement, serialized into text files, and Python scripts that recreate all figures from the text files. [file 41567_2023_2161_MOESM2_ESM.zip › data_and_plotters_all/phase_figures/supfigs/sfig_i_peaks/sfig_i_peaks.pdf]

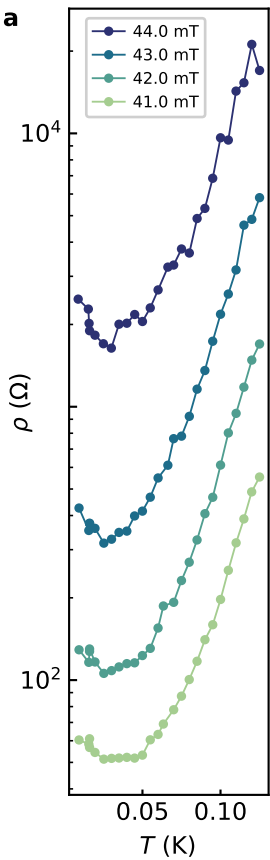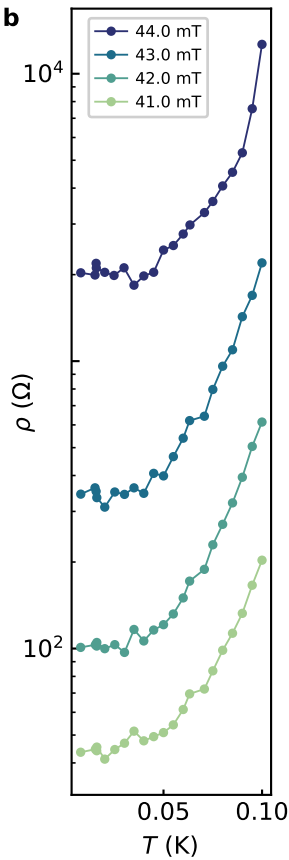

Supplement: Supplementary file 2 — Contains all data in the paper, including supplement, serialized into text files, and Python scripts that recreate all figures from the text files. [file 41567_2023_2161_MOESM2_ESM.zip › data_and_plotters_all/phase_figures/supfigs/sfig_lob_upturn/sfig_lob_upturn.pdf]

**a**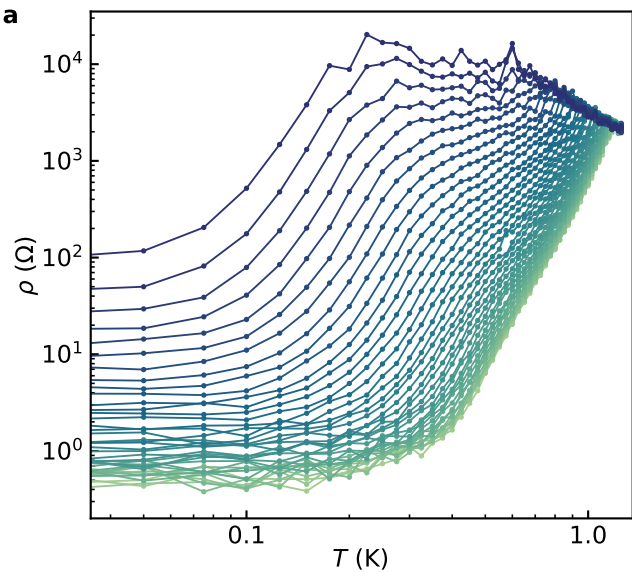**b**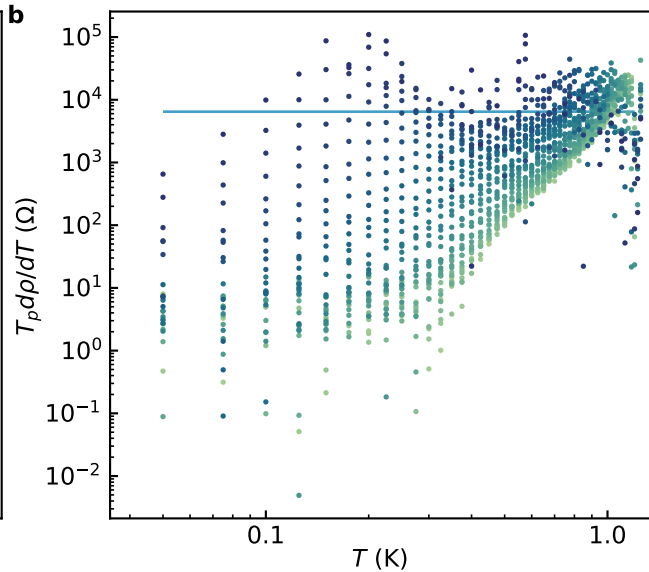

Supplement: Supplementary file 2 — Contains all data in the paper, including supplement, serialized into text files, and Python scripts that recreate all figures from the text files. [file 41567_2023_2161_MOESM2_ESM.zip › data_and_plotters_all/phase_figures/supfigs/sfig_planck/sfig_planck.pdf]

**a**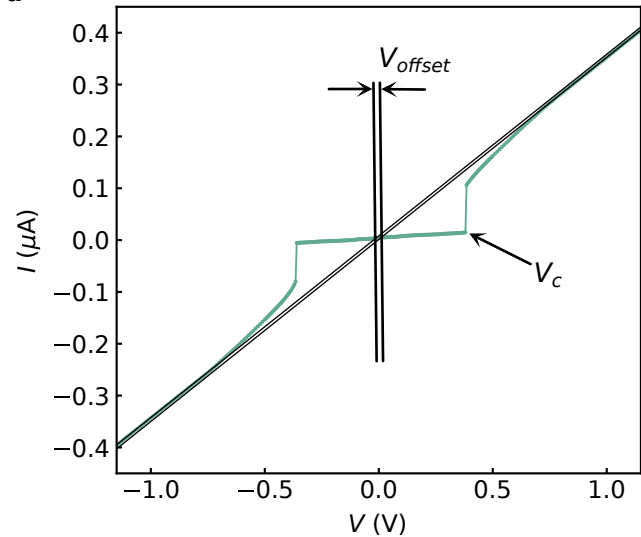**b**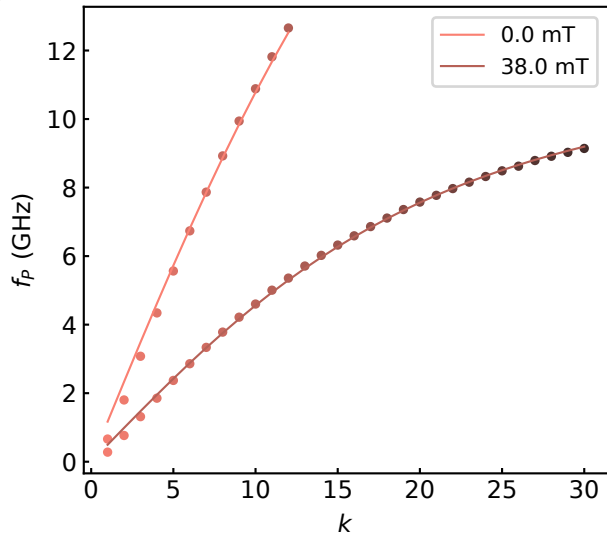

Supplement: Supplementary file 2 — Contains all data in the paper, including supplement, serialized into text files, and Python scripts that recreate all figures from the text files. [file 41567_2023_2161_MOESM2_ESM.zip › data_and_plotters_all/phase_figures/supfigs/sfig_ej_ec_eg/sfig_ej_ec_eg_design.pdf]

**a**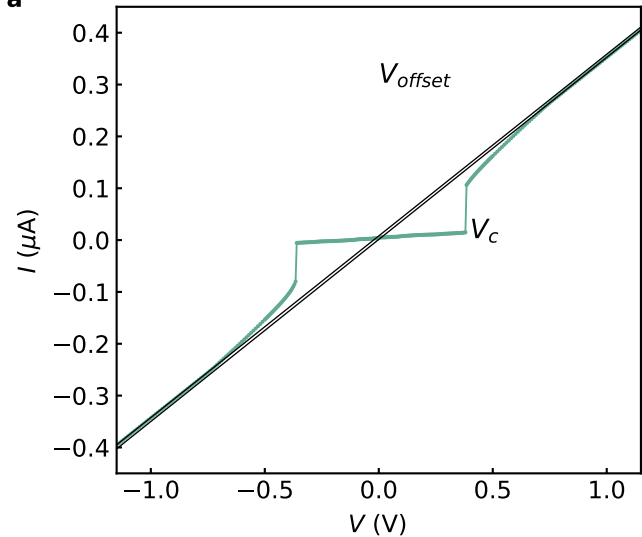**b**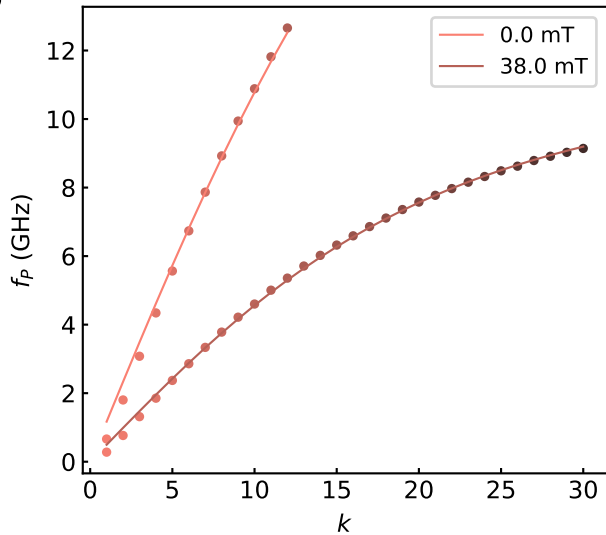

Supplement: Supplementary file 2 — Contains all data in the paper, including supplement, serialized into text files, and Python scripts that recreate all figures from the text files. [file 41567_2023_2161_MOESM2_ESM.zip › data_and_plotters_all/phase_figures/supfigs/sfig_ej_ec_eg/sfig_ej_ec_eg.pdf]

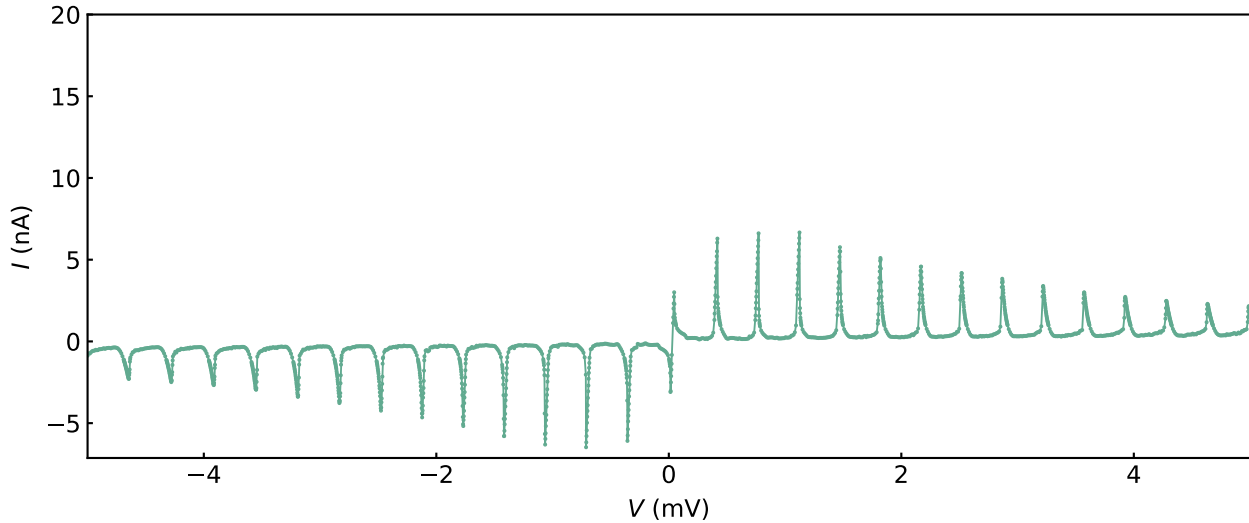

Supplement: Supplementary file 2 — Contains all data in the paper, including supplement, serialized into text files, and Python scripts that recreate all figures from the text files. [file 41567_2023_2161_MOESM2_ESM.zip › data_and_plotters_all/phase_figures/supfigs/sfig_gv_cartoon/sfig_iv_cartoon.pdf]

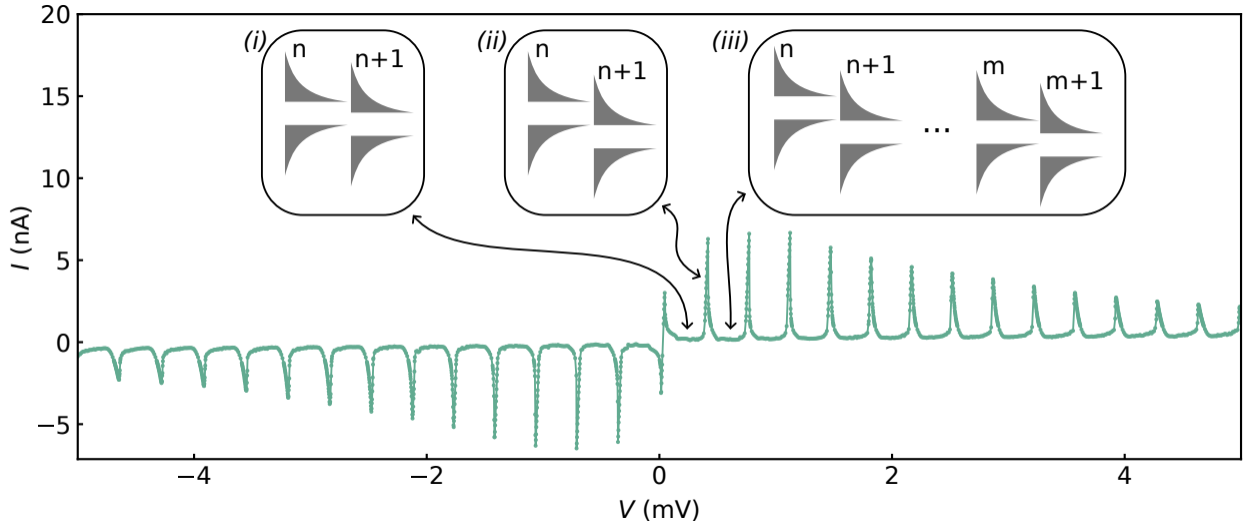

Supplement: Supplementary file 2 — Contains all data in the paper, including supplement, serialized into text files, and Python scripts that recreate all figures from the text files. [file 41567_2023_2161_MOESM2_ESM.zip › data_and_plotters_all/phase_figures/supfigs/sfig_gv_cartoon/sfig_iv_cartoon_design.pdf]

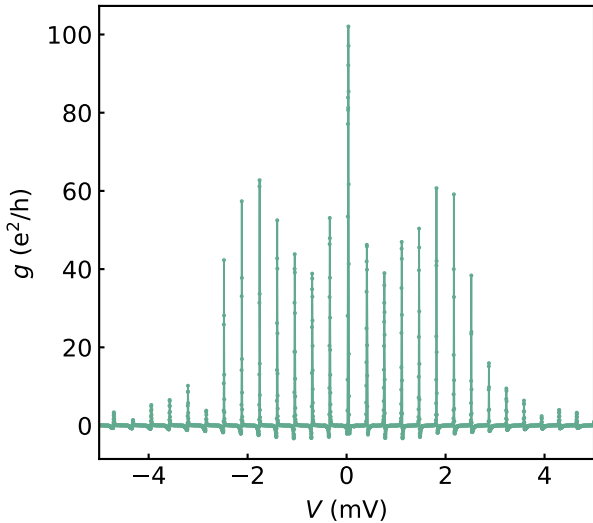

Supplement: Supplementary file 2 — Contains all data in the paper, including supplement, serialized into text files, and Python scripts that recreate all figures from the text files. [file 41567_2023_2161_MOESM2_ESM.zip › data_and_plotters_all/phase_figures/supfigs/sfig_gv_cartoon/sfig_gv.pdf]

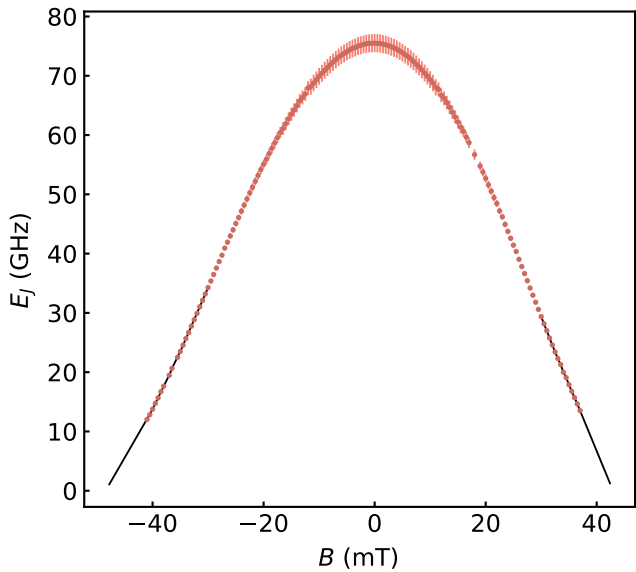

Supplement: Supplementary file 2 — Contains all data in the paper, including supplement, serialized into text files, and Python scripts that recreate all figures from the text files. [file 41567_2023_2161_MOESM2_ESM.zip › data_and_plotters_all/phase_figures/supfigs/sfig_ej_interp/sfig_ej_interp.pdf]

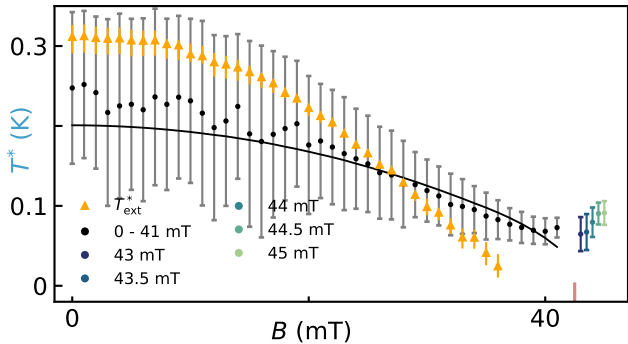

Supplement: Supplementary file 2 — Contains all data in the paper, including supplement, serialized into text files, and Python scripts that recreate all figures from the text files. [file 41567_2023_2161_MOESM2_ESM.zip › data_and_plotters_all/phase_figures/supfigs/sfig_tstar/sfig_tstar_plaw.pdf]

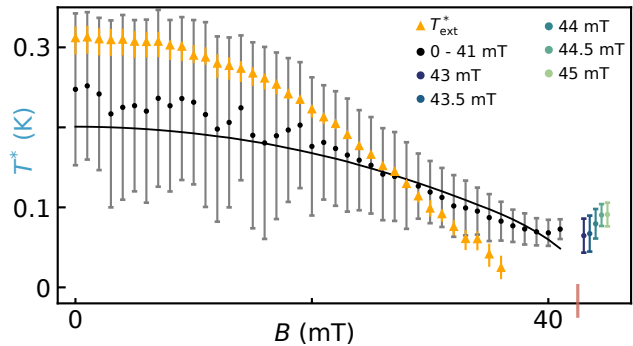

Supplement: Supplementary file 2 — Contains all data in the paper, including supplement, serialized into text files, and Python scripts that recreate all figures from the text files. [file 41567_2023_2161_MOESM2_ESM.zip › data_and_plotters_all/phase_figures/supfigs/sfig_tstar/sfig_tstar_plaw_design.pdf]
